# Role of tubular epithelial arginase-II in renal inflammaging

**DOI:** 10.1038/s41514-021-00057-8

**Published:** 2021-03-02

**Authors:** Ji Huang, Xiujie Liang, Diogo Ladeiras, Benoit Fellay, Xiu-Fen Ming, Zhihong Yang

**Affiliations:** 1 Cardiovascular and Aging Research, Department of Endocrinology, Metabolism, and Cardiovascular System, Faculty of Science and Medicine, University of Fribourg, Fribourg, Switzerland; 2 National Center of Competence in Research “Kidney.CH”, Zürich, Switzerland; 3 Laboratory HFR, Hospital Fribourgeois, Fribourg, Switzerland

**Keywords:** Ageing, Kidney diseases

## Abstract

The aging kidney undergoes complex changes and is vulnerable to injury and development of chronic kidney disease (CKD) with preponderance affecting more women than men. Evidence has been presented that the type-II L-arginine:ureohydrolase, arginase-II (Arg-II) plays a role in the acceleration of aging. Arg-II is highly expressed in the kidney. However, the role of Arg-II in renal aging is not known. This study is to investigate whether Arg-II is involved in the kidney aging process dependently on sex. Arg-II level in the kidney of wild type (WT) mice is significantly elevated with aging, which is accompanied by an increase in expression of the inflammatory cytokines/chemokines, tissue macrophages, factors involved in fibrosis, and tubulointestitial fibrosis in both males and females. This renal aging phenotype is significantly suppressed in *arg-II*^−/−^ mice, mainly in the females in which Arg-II level is higher than in the males. Importantly, numerous factors such as IL-1β, MCP1, VCAM-1, and TGFβ1 are mainly localized in the proximal tubular S3 segment cells expressing Arg-II in the aging kidney. In human proximal tubular cells (HK-2), TNF-α enhances adhesion molecule expression dependently on Arg-II upregulation. Overexpression of Arg-II in the cells enhances TGFβ1 levels which is prevented by mitochondrial ROS inhibition. In summary, our study reveals that renal proximal tubular Arg-II plays an important role in the kidney aging process in females. Arg-II could be a promising therapeutic target for the treatment and prevention of aging-associated kidney diseases.

## Introduction

Aging is an important risk factor of chronic kidney disease (CKD) and is associated with significant structural and functional changes of the kidney in elderly individuals even in the absence of age-related comorbidities^1^. Typically, renal aging is accompanied by the decline of total nephron size and number, global glomerulosclerosis, tubulointerstitial fibrosis involving TGFβ1 and collagen, arteriosclerosis, and inflammation cytokines^2,3^. A combination of two or more of these features is designated by nephrosclerosis^1^. Our understanding of the underlying mechanisms of renal aging is, however, incomplete. As the consequence of steady growth in the population of elderly people in our society, an increased incidence of CKD is predicted for the future^4^. In addition, population-based studies indicate that CKD affects more women than men and women report a higher symptom burden and greater symptom severity than men^5,6^.

Previous studies demonstrate that aging is associated with increased expression of the ureohydrolase type-II or arginase type-II (Arg-II) in different organs including the heart, blood vessels, and pancreas^7–9^. The enzyme metabolizes L-arginine to L-ornithine and urea, resulting in impairment of endothelial function and inflammatory responses due to eNOS-uncoupling and smooth muscle senescence in age-associated vascular dysfunction^10^. Moreover, the enzyme promotes macrophage pro-inflammation responses contributing to accelerated atherosclerosis in *apoE*^−/−^ mouse model and insulin resistance in high-fat-diet (HFD) fed mouse model^11^. In contrast to Arg-I which is mainly expressed in hepatocytes and essential for ammonia detoxification through the urea cycle^12,13^, Arg-II is highly and constitutively expressed in the S3 proximal tubular epithelial cells of the kidney^14^. Yet, the role of Arg-II in CKD remains largely unknown. Convincing evidence shows that Arg-II is not only associated with aging but also plays a causal role in the vascular aging process through a mechanism interacting with mTORC1/S6K pathway^7^. Genetic deficiency in Arg-II in mice reveals protection against atherosclerosis, obesity-linked insulin resistance, and age-related pancreatic beta-cell dysfunction^7,9,11^. It remains, however, unknown whether Arg-II, the major isoform in the kidney, is involved in renal aging phenotype and whether there is a sex-related difference in the renal aging process.

## Results

### Age- and gender-related increase in Arg-II levels in kidney

**Fig. 1:**
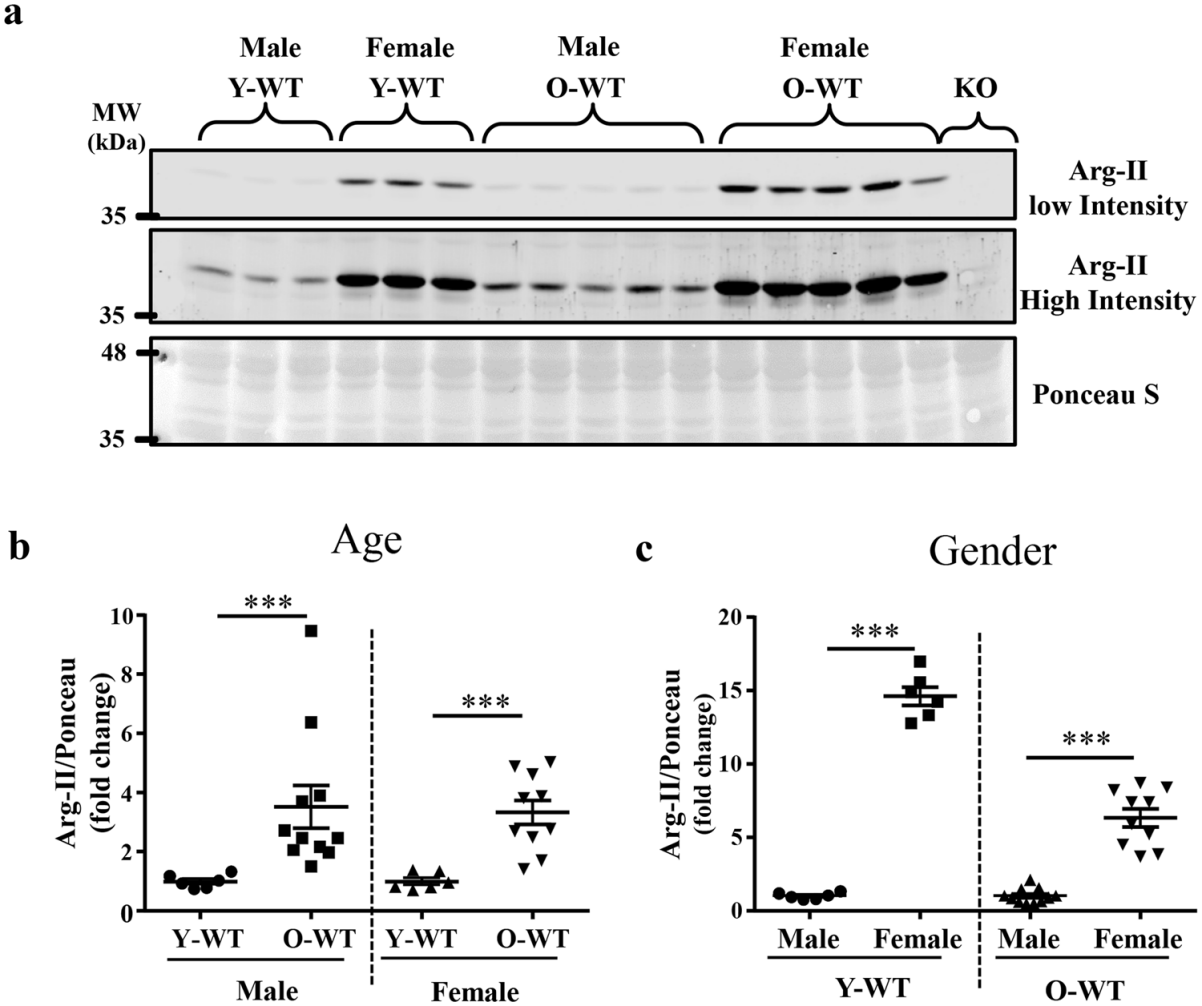
Age and sex-associated differences in arginase-II protein expression. **a** Immunoblotting analyses of arginase-II in kidney of male and female WT mice. Two scanning intensities are shown to better visualize the aging- and gender-associated differences in Arg-II levels (low intensity image was used for female mice and higher intensity image was used for male mice quantification). Kidney from *arg-II*^−/−^ mice was used as a negative control. Ponceau staining serves as loading control, since actin has a variable expression with aging. **b** Quantification is shown in the graphic revealing comparison of young and old mice. **c** Quantification is shown in the graphic revealing comparison of male and female mice. The value of male young and male old mice was used as a reference for female young and female old mice, respectively. Y-WT young WT, O-WT old WT, KO *arg-II*^−/−^. ***p ≤ 0.001 between the indicated groups. Male: Y-WT, *n* = 6; O-WT, *n* = 11; female: Y-WT, *n* = 6; O-WT, *n* = 10.

Immunoblotting analyses demonstrated that Arg-II protein levels were higher in the old WT mice (24–28 months) as compared with young animals (7–8 months) in both male and female groups (Fig. 1a, b). Moreover, the female mice revealed a significantly higher Arg-II level than the male mice at both young and older age (Fig. 1a, c). It is of importance to note that mRNA levels of *arg-II* did not differ among young and old animals either in females or males (Supplementary Fig. 1).

### *Arg-II* deficiency reduces age-associated renal inflammation

**Fig. 2:**
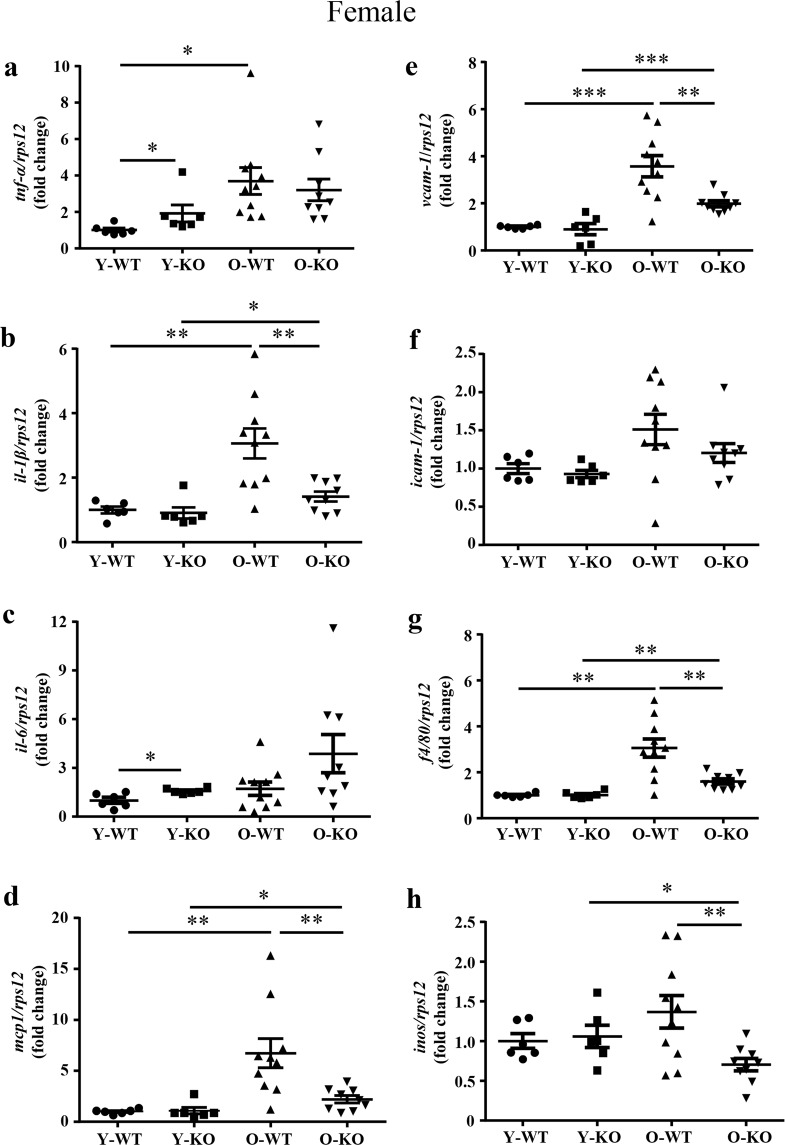
Age-associated inflammation in the kidney was prevented in female *arg-II*^*−/−*^ mice. **a**–**h** mRNA expression levels of *tnf-α, il-1β, il-6, mcp1,vcam-1, icam-1, f4/80* and *inos* in kidney were analyzed by qRT-PCR in female mice. *rps12* served as the reference. Y-WT young WT, Y-KO young *arg-II*^−/−^, O-WT old WT, O-KO old *arg-II*^−/−^. Data are expressed as the fold change to the Y-WT group. *P ≤ 0.05, **P ≤ 0.01, ***P ≤ 0.001. Y-WT, *n* = 6; Y-KO, *n* = 6; O-WT, *n* = 10; O-KO, *n* = 9.

Since aging kidney is associated with inflammation, we then investigated whether the increased Arg-II level plays a role in renal inflammation. For this purpose, the expression of various inflammatory cytokines was analyzed in the kidney of young and old mice. Overall, there was higher expression of *tnf-α*, *il-1β*, *il-6*, *mcp1*, *vcam-1*, *icam-1*, macrophage marker *f4/80*, and *inos* in the old WT mice as compared to the young mice in males (Supplementary Fig. 2a, h) and females (Fig. 2a, h). Of note, this age-associated renal inflammatory phenotype was more pronounced in the females (Fig. 2). *Arg-II* deficiency (*arg-II*^−/−^) significantly reduced *il-1β, mcp1, vcam-1, f4/80*, and *inos* expression in the old female mice (Fig. 2). This effect of *arg-II* deficiency was weaker in the males in which only *il-1β* was significantly reduced (Supplementary Fig. 2b).

### Cellular localization of protein levels inflammatory markers in the aging kidney

**Fig. 3:**
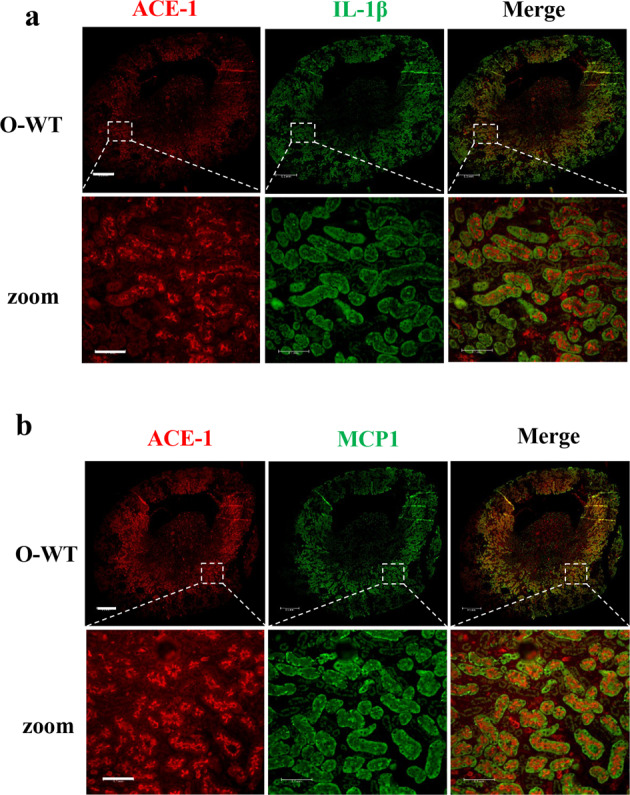
Cellular co-localization of ACE-1/IL-1β and ACE-1/MCP1 in the kidney. Central transverse sections were prepared from old wild type (O-WT) female mice and subjected to immunofluorescence staining of **a** ACE-1 (red) and IL-1β (green) or **b** ACE-1 (red) and MCP1 (green). Enlarged images were selected from outer medulla. Scale bar = 0.5 mm for upper panels in (**a**, **b**) and 0.1 mm for lower panels in (**a**, **b**).

**Fig. 4:**
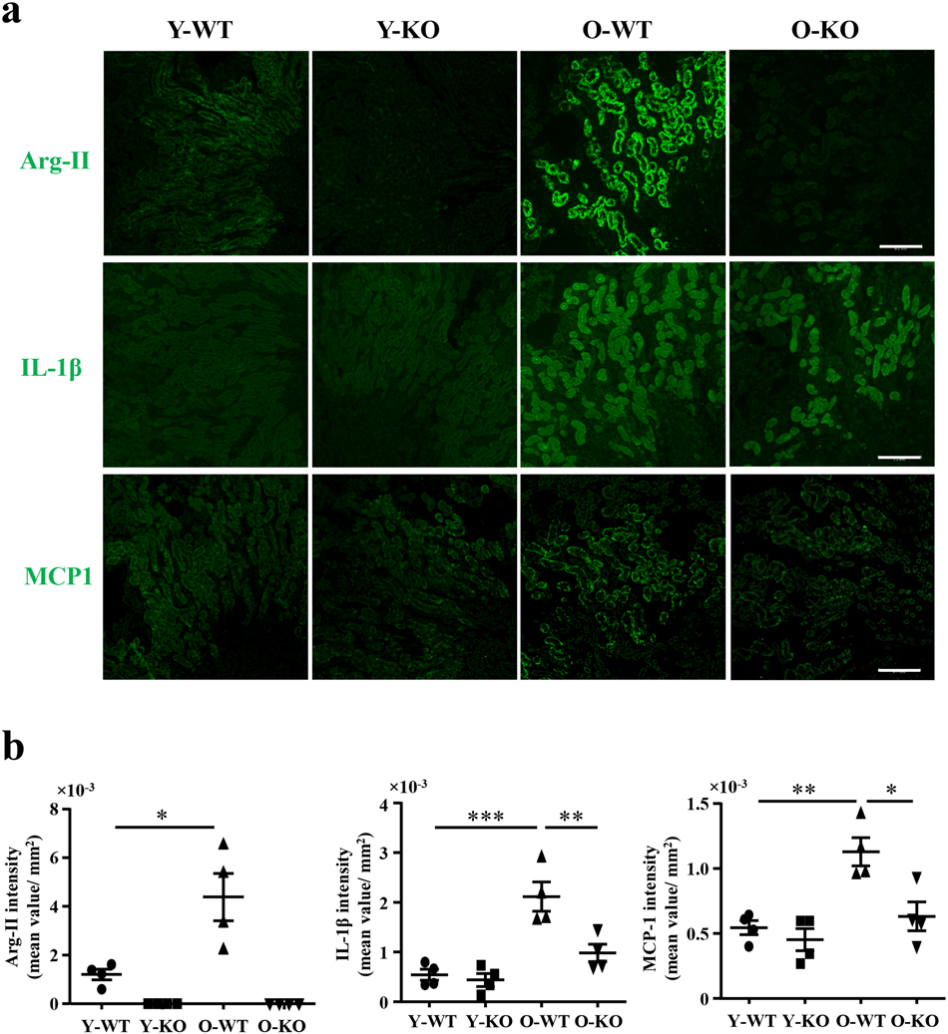
*Arg-II* deficiency reduces age-associated IL-1β and MCP1 in the mouse kidney. Renal paraffin sections (one section from each animal) were prepared from young and old wild type (WT) and *arg-II*^−/−^ female mice (*n* = 4 in each group) and subjected to immunofluorescence staining of **a** Arg-II, IL-1β and MCP1, respectively. Representative images were selected from the outer medulla. Scale bars = 0.2 mm. **b** Quantification of the intensity of fluorescence signals from the whole renal section area was performed with LAS X software. *P ≤ 0.05, **P ≤ 0.01, ***P ≤ 0.001. Y-WT young WT, Y-KO young *arg-II*^−/−^, O-WT old WT, O-KO old *arg-II*^*−/−*^.

**Fig. 5:**
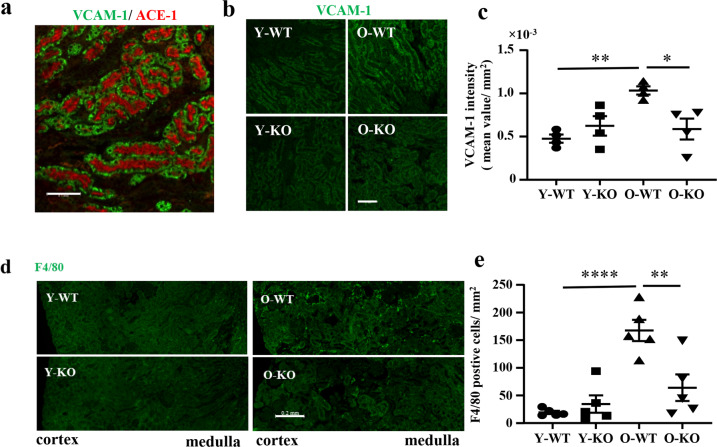
*Arg-II* deficiency reduces age-associated VCAM-1 expression and macrophage accumulation in female mice. Central transverse renal sections (one section from each animal) were prepared from young and old WT and *arg-II*^−/−^ female mice and subjected to immunofluorescence staining of **a** VCAM-1 and ACE-1 from an old WT female mouse (left panel), **b** VCAM-1 (right panel), *n* = 4; and **d** F4/80 (*n* = 5). Representative images for ACE-1 and VCAM-1 were selected from outer medulla. Scale bars = 0.1 mm. The representative images for F4/80 contain cortex and medulla. Scale bars =;0.2 mm. **c**, **e** Quantification of the intensity of fluorescence signals from the whole renal section area was performed with LAS X software. *P ≤ 0.05, **P ≤ 0.01, ****P ≤ 0.0001. Y-WT young WT, Y-KO young *arg-II*^−/−^, O-WT old WT, O-KO old *arg-II*^−/−^.

Since the age-associated renal inflammatory phenotypes and inhibition by *arg-II* deficiency are more pronounced in female mice, cellular localization of the most relevant inflammatory cytokines, i.e., IL-1β, MCP1, VCAM-1, and F4/80 in the kidney was further investigated in females. Since angiotensin-converting enzyme-1 (ACE-1) and Arg-II both are exclusively expressed in the S3 segment of renal proximal tubules under physiological condition^15,16^; they can be used as S3 segment markers for co-staining with the inflammatory cytokines. Because all the antibodies for the above-mentioned cytokines and Arg-II are from rabbit and anti-ACE-1 antibody is from mouse, cellular localization of the cytokines in the S3 segment was analyzed by co-staining with ACE-1. Immunofluorescence microscopy results showed that IL-1β and MCP1 were mainly localized in ACE-1-expressing cells (Fig. 3a, b), i.e., mainly and abundantly expressed in the S3 segment, although they were also present in some of the other cells or other parts of the kidneys. Single staining of the molecules and quantification of immunofluorescence signals demonstrated elevated Arg-II signal in the S3 segment of old than young WT mouse, while no signal of Arg-II staining could be detected in the *arg-II*^−/−^ mice as expected (Fig. 4a, b). The results confirmed the findings on Western blotting shown in Fig. 1a. Furthermore, augmented IL-1β and MCP1 levels were found in old mice as compared to the young mice, which was significantly reduced in the age-matched *arg-II*^−/−^ animals (Fig. 4a, b). In addition, VCAM-1 was highly expressed in the S3 segment of old mice as evidenced by co-staining with apical ACE1-expressing tubular epithelial cells (Fig. 5a). The age-associated increase in VCAM-1 staining was decreased in age-matched *arg-II*^−/−^ mice (Fig. 5b, c). Furthermore, there was a substantial macrophage accumulation in the aging kidney as demonstrated by F4/80 staining from cortex to medulla (Fig. 5d), which was markedly reduced in the *arg-II*^−/−^ mice (Fig. 5d, e).

### *Arg-II* deficiency reduces age-related renal fibrosis and TGF-β1

**Fig. 6:**
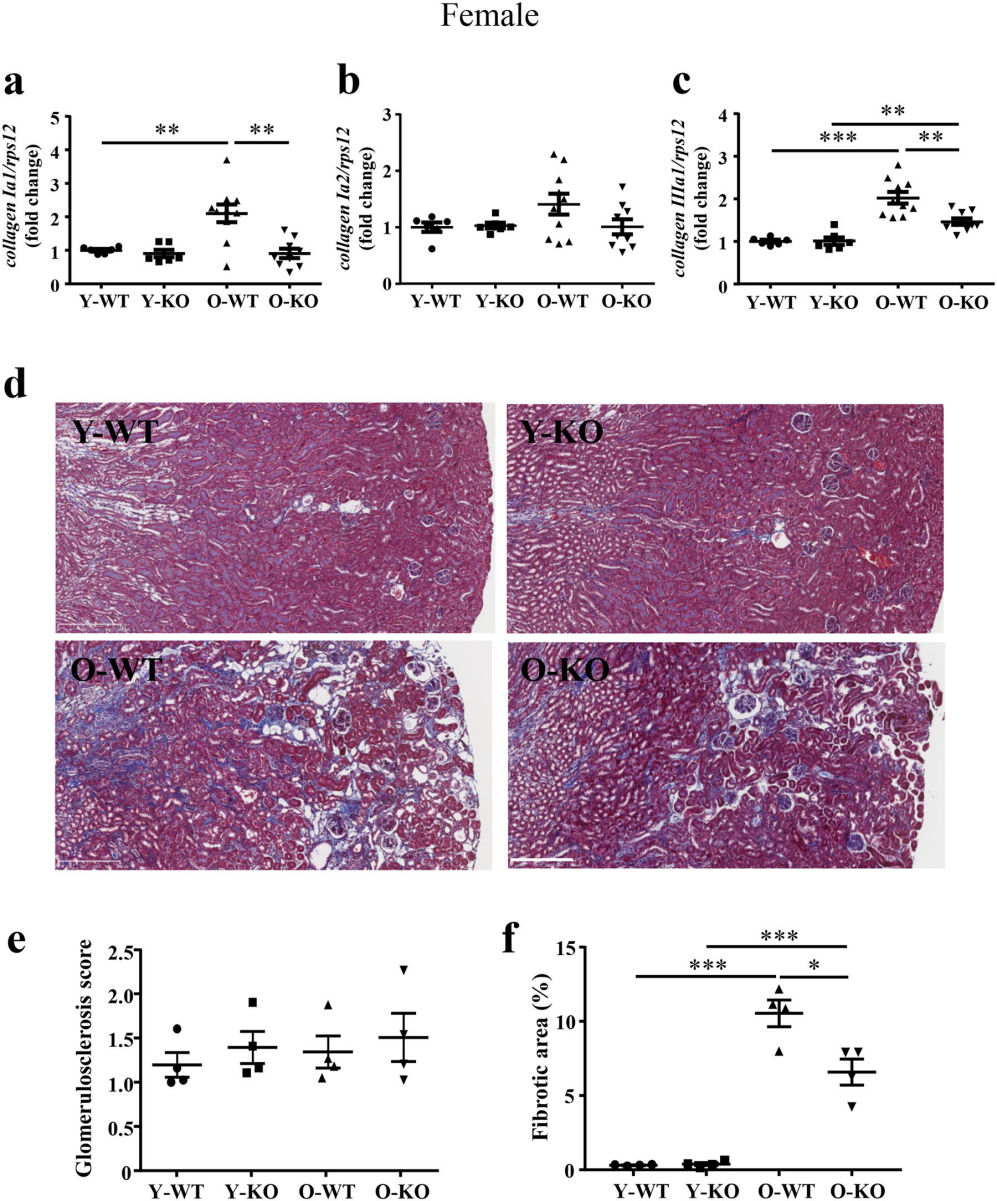
Age-associated increase in fibrosis is reduced by *arg-II* deficiency in female mice. **a**–**c** mRNA expression levels of *collagen type Iα1, Ia2*, and *IIIa1* were analyzed by qRT-PCR in female mice, respectively. *rps12* was used as a reference. Data are expressed as the fold change to Y-WT group. Y-WT, *n* = 6; Y-KO, *n* = 6; O-WT, *n* = 10; O-KO, *n* = 9. **d** Representative histological images (magnification ×10) of Masson trichrome stain in young and old WT and *arg-II*^−/−^ female mice. Scale bar = 250 μm. *n* = 4 in each group. **e** Quantification of glomerulosclerosis was represented by glomerulosclerosis scores (see method section) and **f** interstitial fibrosis was shown by the percentage of renal fibrotic area. Y-WT young WT, Y-KO young *arg-II*^−/−^, O-WT old WT, O-KO old *arg-II*^−/−^. *P ≤ 0.05, **P ≤ 0.01, ***P ≤ 0.001.

**Fig. 7:**
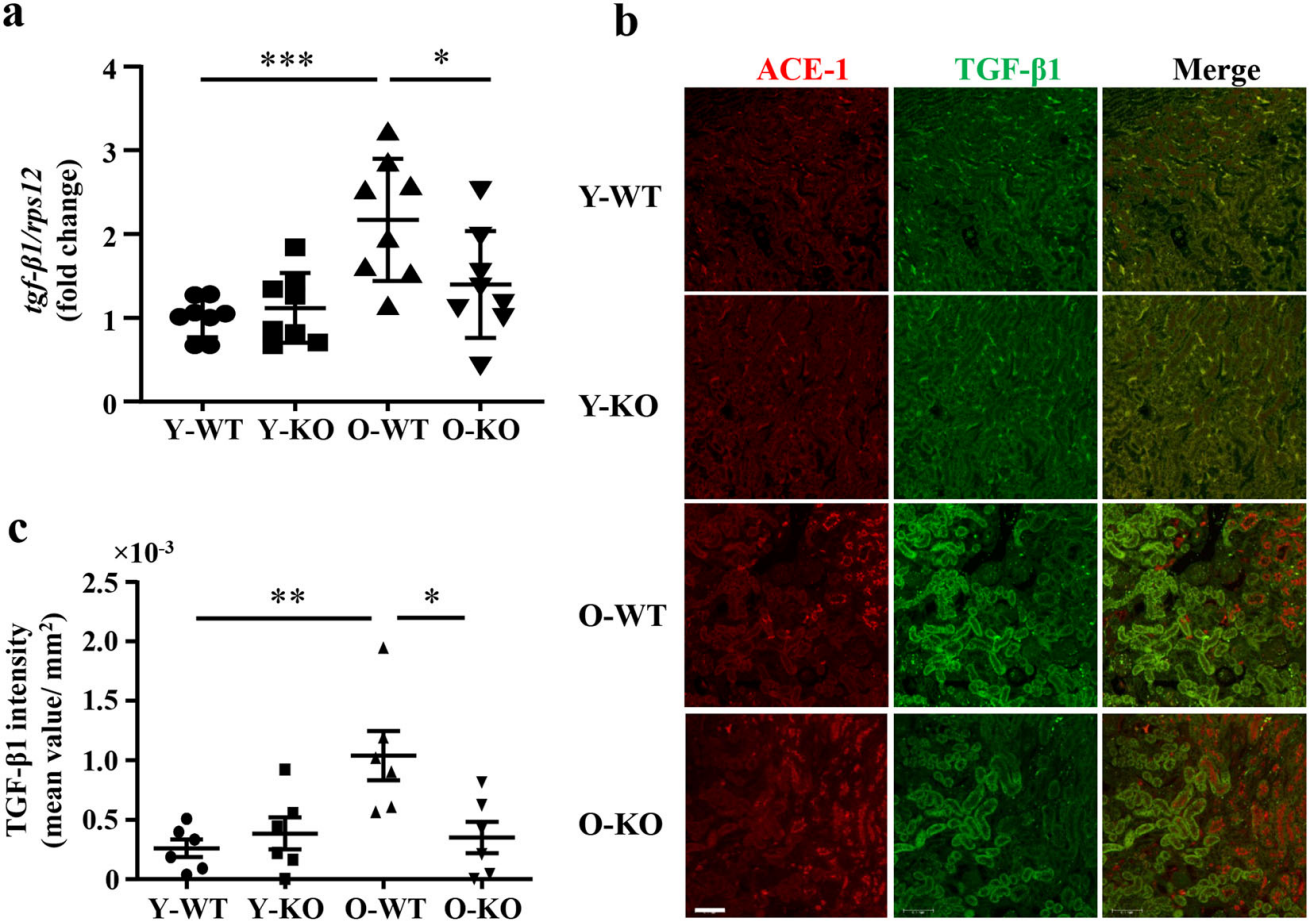
*Arg-II* deficiency reduces age-associated TGF-β1 in female mice. **a** mRNA levels of *tgf-β1* in young and old WT and *arg-II*^−/−^ mouse kidneys analyzed by qRT-PCR; *n* = 8 in each group; **b** Central transverse renal sections (one section from each animal) were prepared from young and old WT and *arg-II*^−/−^ female mice and subjected to immunofluorescence staining of ACE-1 (red) and TGF-β1 (green). Representative images were selected from the outer medulla. *n* = 6 in each group. Scale bars = 0.1 mm. **c** Quantification of the intensity of fluorescence signals of TGF-β1. Y-WT young WT, Y-KO young *arg-II*^−/−^, O-WT old WT, O-KO old *arg-II*^−/−^. *P ≤ 0.05, **P ≤ 0.01, ***P ≤ 0.001.

We found an age-associated increase in *collagen Iα1* and *collagen IIIα1* in the kidney of female mice (Fig. 6a, c) and associated renal peritubular interstitial fibrosis (Fig. 6d). This phenotype was not present in the male group (Supplementary Fig. 3). The age-associated increase in *collagen* expression and renal peritubular interstitial fibrosis in females was significantly reduced in the *arg-II*^−/−^ mice (Fig. 6a, c, d, f). No difference in glomerulosclerosis was observed among the groups (Fig. 6e). In the aging kidneys of females, TGF-β1 mRNA and protein levels were enhanced as compared to the young mice, which was reduced in the age-matched *arg-II*^−/−^ mice (Fig. 7a–c). As shown in Fig. 7b, TGF-β1 was localized in proximal tubular cells, including the ACE1-expressing S3 segment tubular cells.

### Silencing *arg-II* inhibits inflammation in human proximal tubular epithelial cells

**Fig. 8:**
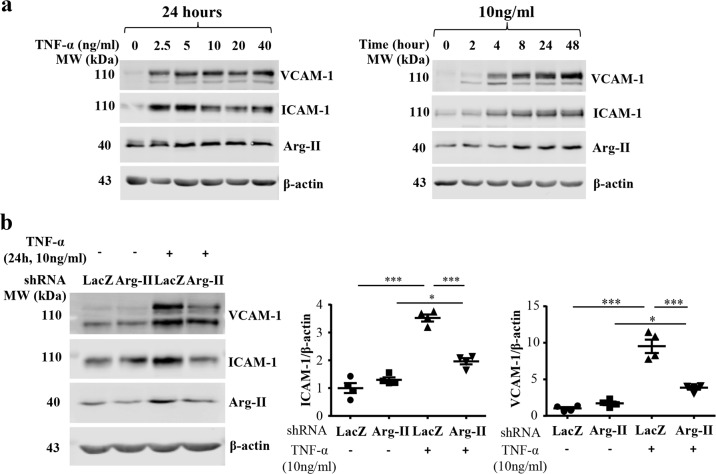
Silencing *arg-II* decreases TNF-α induced expression of adhesion molecules in HK-2 cells. **a** HK-2 cells were serum starved for 24 h, then incubated in the presence of various concentration or various duration of TNF-α. Cell lysates were prepared and subjected to immunoblotting analysis of VCAM-1, ICAM-1, Arg-II, and β-actin. β-actin served as loading control. **b** HK2 cells were transduced with rAd/U6-LacZ^shRNA^ as control (LacZ) or rAd/U6-Arg-II^shRNA^ to silence *arg-II* gene (*arg-II*). 2 days post-transduction, cells were serum starved for 24 h, then incubated with 10 ng/ml TNF-α for 24 h. Cell lysates were prepared and subjected to immunoblotting analysis of VCAM-1, ICAM-1, Arg-II, and β-actin. Data are presented as mean ± SEM. *n* = 4. *P ≤ 0.05, ***P ≤ 0.001. rAd recombinant adenovirus.

To confirm the role of proximal tubular epithelial cell Arg-II in inflammation responses, human HK-2 cells were cultured and stimulated with TNF-α. As shown in Fig. 8a, TNF-α enhanced VCAM-1 and ICAM-1 as well as Arg-II protein levels (but not mRNA levels, Supplementary Fig. 4) in a concentration-dependent and time-dependent manner. Silencing *arg-II* gene markedly reduced TNF-α (10 ng/ml, 24 h)-induced ICAM-1 and VCAM-1 levels in the cells (Fig. 8b), demonstrating that Arg-II indeed mediates inflammation in the proximal epithelial cells.

### Role of mitochondrial oxidative stress in Arg-II-induced TGF-β1 production in human proximal tubular epithelial cells

**Fig. 9:**
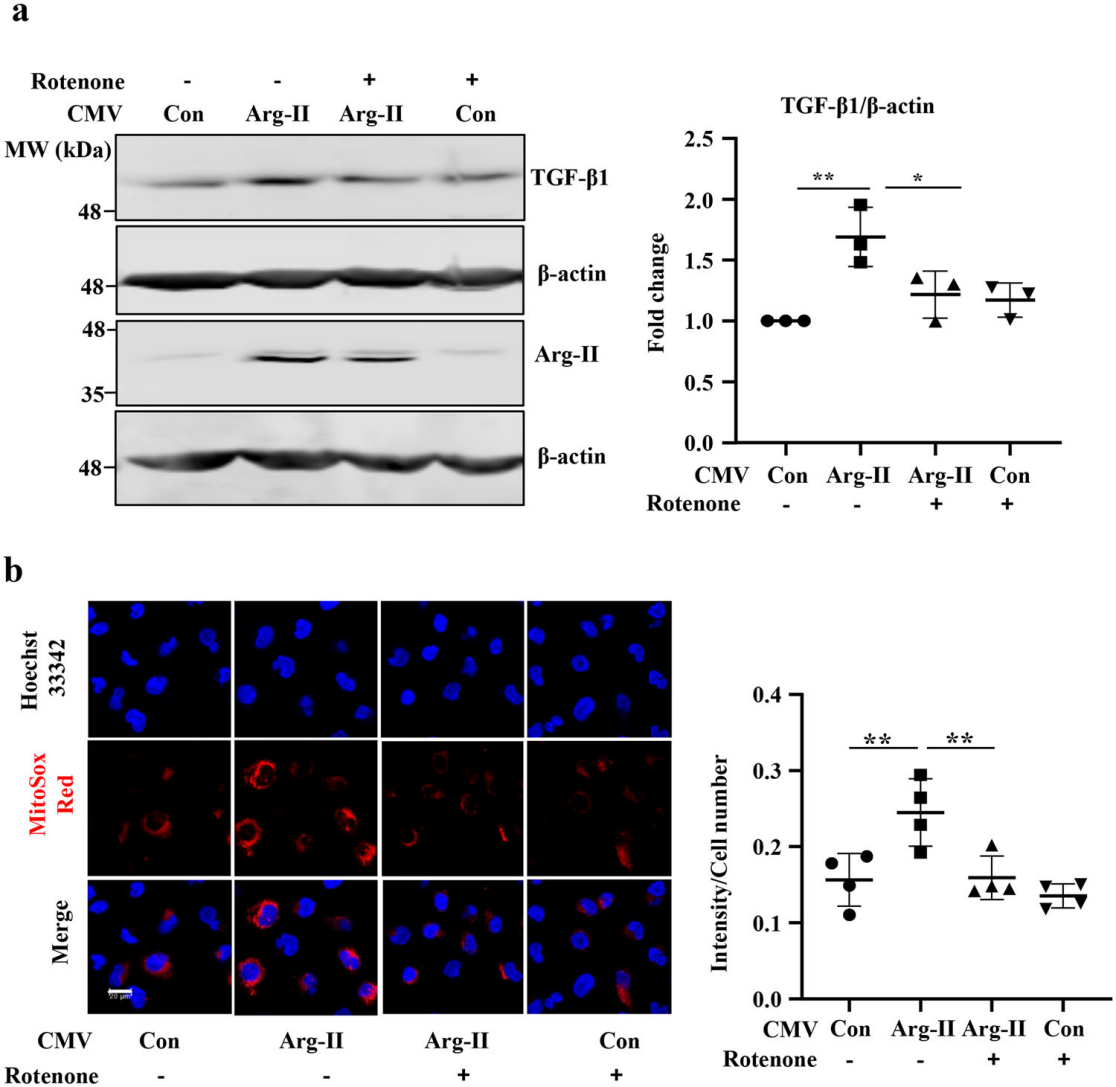
Arg-II induces TGF-β1 expression via mitochondrial oxidative stress in HK-2 cells. HK-2 cells were pre-treated with rotenone (2 μmol/L) for 1 h and then were transduced with rAd/CMV empty vector as control (V5) or rAd/CMV-Arg-II (Arg-II) for 24 h. **a** Immunoblotting analysis for TGF-β1, Arg-II, and β-actin. The graph on the right shows the quantification of the signals on immunoblots. *n* = 3. **b** Cells were subjected to mitochondrial superoxide detection with MitoSox (red), followed by nuclei staining with Hoechst 33342 (blue). The images were taken from MitoSox staining through ×40 objectives with Leica TCS SP5 confocal microscope. *n* = 4. Scale bar = 20 μm. The plot graphs on the right show the quantification of the signals. Con control. Data are presented as mean ± SEM. *p ≤ 0.05, **p ≤ 0.01.

In HK2 cells, overexpression of *arg-II* enhanced TGF-β1 protein level which was inhibited by rotenone (2 μmol/L), an inhibitor of mitochondrial respiration chain complex-I (Fig. 9a). The mitochondrial ROS production as demonstrated by MitoSox staining was enhanced in the cells with *arg-II* overexpression, which was inhibited by rotenone (Fig. 9b). The results demonstrate that overexpression in of *arg-II* in the proximal tubular cells enhances TGF-β1 production involving mitochondrial ROS.

### Cell apoptosis and proliferation in aging kidney

**Fig. 10:**
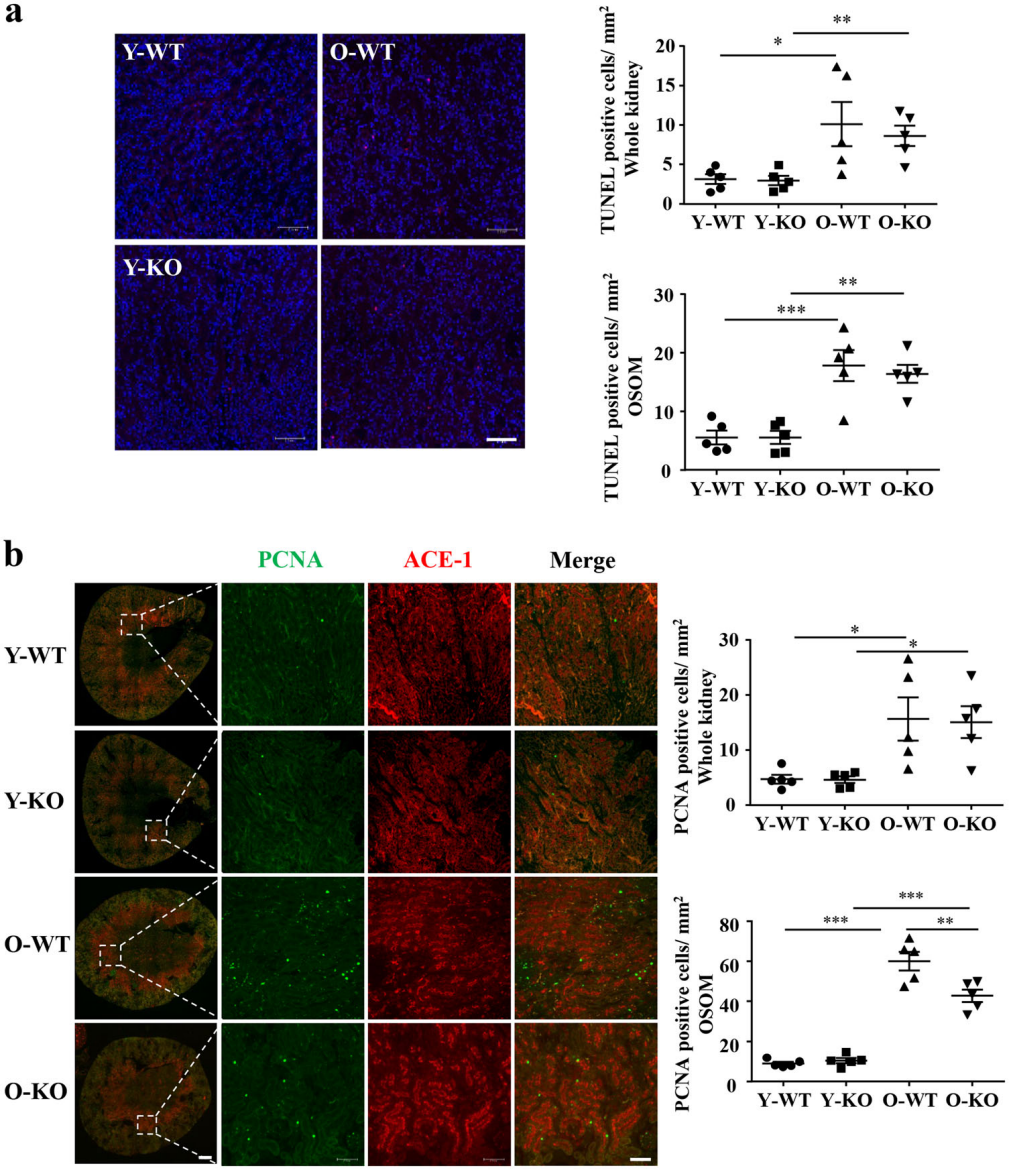
Age-associated proliferation, but not apoptosis, in outer strip of outer medulla was alleviated by *arg-II* deficiency. Central transverse renal sections (one section from each animal) were prepared from young and old WT and *arg-II*^−/−^ female mice. **a** TUNEL assay of apoptotic cells (red) in renal paraffin section. Quantification of TUNEL positive cells per mm^2^ in the whole kidney section and outer strip of outer medulla (OSOM) delimited by ACE-1 (marker of S3 segment of proximal tubule) positive area in adjacent kidney section is presented as dot plots in the right panels. Scale bars = 0.1 mm. n = 5. **b** Immunofluorescence staining of PCNA (green, a marker of proliferating cells) and ACE-1 (red). The right images are the enlargements of the selected area in the corresponding pictures immediately to the left. Scale bars = 0.5 mm in the left panels and 0.1 mm in the right panels, respectively. Quantification of PCNA-positive cells per mm^2^ in the whole kidney section and OSOM which is delimited by ACE-1 positive area is presented as dot plots in the right panels, *n* = 5. Y-WT young WT, Y-KO young *arg-II*^−/−^, O-WT old WT, O-KO old *arg-II*^−/−^. *P ≤ 0.05, **P ≤ 0.01, ***P ≤ 0.001.

As shown by TUNEL staining and PCNA staining, both cell apoptosis and cell proliferation in the whole kidney were enhanced in the aging, particularly in the outer strip of the outer medullar (Fig. 10a, b). Of note, a significant portion of the cell proliferation occurred in cells expressing ACE1, another marker of S3 segment (Fig. 10b). The cell proliferation but not apoptosis in the outer strip of the outer medullar in the aging kidney were significantly reduced by *arg-II*^−/−^ (Fig. 10a, b).

### Age-associated increase in p16^INK4a^ and mTORC1-S6K1 signaling in the kidney

The aging marker *p16*^*INK4a*^ was not detectable in young animals (Supplementary Fig. 5a, c). An increased expression of *p16*^*INK4a*^ in the aging kidney of both male and female mice was observed in the females, which is however not significantly affected by *arg-II* gene deficiency (Supplementary Fig. 5a, c). An age-associated increase in S6K1 signaling as assessed by phosphorylation of ribosomal S6 at serine 240/244 in female mice was observed, and this increased S6K signaling tended to be decreased in the *arg-II*^*−/−*^ mice, but did not reach statistical significance (Supplementary Fig. 5b, d). These aging markers were further confirmed by confocal immunofluorescence staining of p16^INK4α^ and pS6K1. The results show that the age-associated increase in p16^INK4α^ staining in old female mouse kidneys either in the cortex or medullar was not significantly affected by *arg-II*^−/−^ (Supplementary Fig. 6). Also the level of pT389-S6K1 was enhanced in aging but not affected by *arg-II*^−/−^ (Supplementary Fig. 7).

## Discussion

In this study, we report that renal Arg-II expression is augmented in proximal tubular S3 segment cells with aging in a mouse model and ablation of Arg-II reduces renal inflammaging phenotypes mainly in the female mice. This finding implicates that elevated Arg-II plays a causal role in accelerating the renal aging process.

Previous studies showed that Arg-II is upregulated in various organs, including the heart, blood vessels, skin, and pancreas in aging^7–9^. Ablation of *arg-II* globally extends lifespan and protects mice from organ dysfunction in aging and age-associated chronic diseases^8^. For example, *arg-II*^−/−^ mice reveal preserved vascular endothelial function in aging, in high-fat-diet and high-cholesterol-diet induced atherosclerosis and obesity models and improved glucose tolerance in obesity and aging^7–9^. The beneficial effects of *arg-II* deficiency in these models are due to inhibition of eNOS-coupling, reduced macrophage pro-inflammatory responses and systemic inflammation, and preservation of pancreatic β-cell function^7–9^. Similar to those organs, our current study shows that enhanced protein levels of Arg-II also occur in the aging kidney in the S3 segment of proximal tubules. The aging kidney phenotype is significantly blunted by *arg-II* deficiency, which is in line with the previous findings on the role of *arg-II* in accelerating the aging process^7–9^. It is of note that the female mice have higher Arg-II protein levels than the male mice and under physiological condition the young females have higher Arg-II than the old males. The high expression of Arg-II in young female mice is most likely physiological, though the physiological relevance remains unknown. The observation that the young female mice have higher Arg-II levels than the old males without aging phenotype suggests that Arg-II expression in the kidney alone is not sufficient to drive kidney dysfunction under physiological conditions. It is understandable that Arg-II is not the only factor that drives the aging process. The aging phenotype becomes obvious only in the later life, if Arg-II is upregulated in conjunction with other age-associated changes during life time. The fact that *arg-II*^−/−^ dampens the kidney aging phenotype demonstrates that elevated Arg-II in the kidney importantly contributes to kidney aging, although it alone is not sufficient to drive kidney dysfunction.

It is of note that there is no difference in *arg-II* mRNA expression with aging in the kidney of both males and females despite elevated protein levels with aging. This observation implicates that age-associated increase in Arg-II protein level is regulated at the posttranscriptional and/or translational level. This conclusion is further supported by the experiments in a human proximal tubular cell line, HK-2 cells which are treated with the inflammatory cytokine TNFα that was found to be elevated in the aging kidneys. As shown in Fig. 8, TNFα increases Arg-II protein levels, while it decreases its mRNA levels in the cells. Moreover, TNFα increases VCAM-1 and ICAM-1 protein levels which are prevented by silencing *arg-II* in the HK2 cells. The results indicate that elevated Arg-II is regulated in the aging kidney at the posttranscriptional and/or translational level in proximal tubular cells and contributes to inflammatory responses. The results also suggest that there is a vicious cycle between inflammation and Arg-II. Detailed mechanisms of Arg-II regulation in aging remain to be investigated.

As shown there is a gender difference in renal Arg-II expression. In contrast to the lack of the effect of aging on *arg-II* mRNA levels, there is a gender effect on mRNA levels, i.e., the renal *arg-II* expression including mRNA and protein levels is higher in females than the males either in the young or in aged group, which is consistent with the reports in the literature^14,17^. This gender difference in Arg-II expression is also observed in other tissues, including the pancreas, skin, and heart^8,9^. Although the mechanisms involved are currently unknown, a direct hormonal effect may not explain the phenomenon, since estradiol has been shown to attenuate Arg-II expression^18,19^ and testosterone induces its levels in the kidney^17^. The higher renal Arg-II levels in females may explain the stronger effect of *arg-II* deficiency on renal inflammaging phenotypes in this gender of mice. Indeed, we observed a significant enhancement of several pro-inflammatory cytokines/chemokine and macrophages in the kidney of old female mice. The majority of the inflammatory responses are reduced in *arg-II*^−/−^. These findings are in line with the studies showing that Arg-II plays a role in inflammation in other models or tissues as discussed above, demonstrating a pro-inflammatory role of augmented Arg-II in several chronic inflammatory disease conditions and in aging^9,15,20^.

Since mTORC1/S6K1 pathway plays an important role in cellular senescence and organismal aging^21,22^ and in the vasculature, there is a positive mutual regulation between Arg-II and S6K1 pathway which plays a causal role in vascular endothelial cell aging^7^. A possible interaction of Arg-II and S6K1 was analyzed in the kidney. The renal S6K1 activation as analyzed by the levels of phosphor-S6K1 and p-S6 substrate was significantly increased in old female mice as compared to the young animals, which is not affected by *arg-II* deficiency. These results suggest that Arg-II does not interact with the S6K1 signaling pathway in kidney aging.

One of the remarkable characteristics of renal aging is tubulointerstitial fibrosis^23,24^. We find enhanced *collagen Iα1, collagen IIIα1* expression, and renal tubulointerstitial fibrosis of old mice which is reduced in the age-matched *arg-II*^−/−^ animals. Since Arg-II metabolizes L-arginine to L-ornithine, leading to a more pronounced synthesis of L-proline, the precursor for collagen production^25^. This mechanism could partly explain enhanced tubulointerstitial fibrosis in the aging kidney, which is reduced when *arg-II* gene is disrupted in female mice. In line with our findings, Morris and colleagues have demonstrated that *arg-II*^−/−^ mice had decreased renal fibronectin expression in diabetic nephropathy^26^, which supports the role of Arg-II in the development of renal fibrosis. The partial inhibition of renal fibrosis and lack of significant effect of *arg-II* deficiency in male mice suggest that other mechanisms are also involved, which requires further investigation. It has been shown that inflammation and fibrosis intertwine with each other and promote renal aging phenotype^2,27^. Our mouse renal aging model shows similar phenotypes of inflammation and fibrosis as in humans. The database of a transcriptional profile study of human kidney aging shows that renal expression of *vcam-1, mcp1, collagen α1*, and *collagen IIIα1* are upregulated in aging^28^. The results of our study are in line with this concept and demonstrate a role of Arg-II in promoting renal aging phenotype related to inflammation and fibrosis. One of the factors that play an important role in renal fibrosis is TGFβ1 which is considered the key driver of renal fibrosis^29^. In our current study, we showed that elevated TGFβ1 levels are found in the proximal tubular cells including S3 segment cells expressing ACE-1 and Arg-II in old mice and *arg-II* deficiency decreased renal TGFβ1. In addition, overexpression of Arg-II in HK-2 cells enhances TGFβ1 levels and causes mitochondrial ROS production and inhibition of mitochondrial ROS prevents TGFβ1 production by Arg-II. These results strongly suggest that elevated Arg-II in renal tubular cells increases TGFβ1 expression through mitochondrial oxidative stress, contributing to renal fibrosis in aging.

It is also interesting to note that a more pronounced cell proliferation marker PCNA was observed in the outer strip of out medulla of old mice than in the young mice, which was significantly reduced by *arg-II* deficiency. The PCNA positive cells are located in the tubular epithelial cells as well as non-epithelial cells. The enhanced PCNA positive cells have been shown to reflect dedifferentiation of the proximal tubule epithelial cells or proliferative progenitor cells and/or myofibroblasts in response to injury to counteract the insults^30,31^. The less PCNA positive cells in the old *arg-II*^−/−^ mice as compared to the age-matched WT controls could be well explained by less renal injury in the former group. This conclusion is well supported by the fact that *arg-II*^−/−^ mice reveal less inflammaging markers in the aging kidney than the WT controls. In contrast to the proliferation marker, no difference in cell apoptosis could be found between WT and *arg-II*^−/−^ mice in aging, suggesting that Arg-II does not have a significant impact on the regulation of cell apoptosis in the aging kidney.

It is to note that a recent published study with conditional renal *arg-II* knockout mouse reported a protective role of Arg-II in the acute ischemia-reperfusion injury in mice^32^, which contrasts to the majority of the literature and to the findings in our current study. There are several reasons which may explain the discrepant findings. Firstly, we appreciate that conditional renal and systemic *arg-II* knockout mice are used between their and our study. Secondly, they used the acute renal ischemia-reperfusion injury model, while we studied age-associated renal damage, a chronic CKD model. The underlying mechanisms shall be different. During preparation of our manuscript, we appreciate that another recently published study by Hara and colleagues demonstrated a detrimental role of Arg-II in mediating ischemia-reperfusion injury in the kidney^33^, which is in accordance with our current study showing that Arg-II, if it is upregulated, promotes renal inflammation and injury. Although there is a concern that the renal Arg-II’s protective role could be masked by Arg-II and/or Arg-I in the extra-renal tissues in the systemic *arg-II*^−/−^ model, the findings of our study that enhanced Arg-II expression in S3 segment epithelial cells in aging is highly accompanied with increased expression and production of several pro-inflammatory cytokines/chemokines and adhesion molecules in this particular cells, and *arg-II*^−/−^ reduced these phenotypes associated with aging, support a role of Arg-II as a mediator of renal damage in CKD. This conclusion is also supported by the results obtained from cultured human proximal epithelial HK-2 cells, in which TNF-α enhances adhesion molecule expression in parallel with upregulation of Arg-II, and silencing *arg-II* markedly reduced the inflammatory responses in the cells. These results demonstrate a pivotal role of proximal tubular epithelial cell Arg-II in mediating inflammation or inflammaging observed in the aging kidney model of our study and also in ischemia-reperfusion injury model^33^. This concept is in line with the recognition that tubular epithelial cell injury plays a central role in CKD^30,34,35^. Despite these findings, our current study does not exclude the possibility that the protective effects of systemic *arg-II* deficiency on renal aging could be contributed secondarily by other tissues, organs, and cells that express *arg-II*. The future study shall confirm our findings in renal specific Arg-II knockout aging mouse model and also in humans.

Despite the limitations of the mouse models, the results of our study implicate that elevated Arg-II in the kidney with aging plays an important role in renal inflammaging with the preponderance in females. Genetic ablation of *arg-II* reduces the renal inflammaging phenotypes and may represent a therapeutic target to retard the renal aging process and/or age-related chronic renal diseases.

## Methods

### Materials

Reagents were purchased from the following sources: Mouse antibody against S6 (#2317s) and rabbit antibody against Arg-II (#55003), phospho S6-S240/244 (#5364), F4/80 (#30325S), PCNA (#13110) and Phospho-p70 S6 Kinase-Thr389 (#9234) were purchased from Cell Signaling Technology (Danvers, USA); Mouse antibody against ACE1 (sc-23908), ICAM-1 (sc-8439) and p16 (sc-81156) were from Santa Cruz Biotechnology (Dallas, USA); Rabbit antibody against IL-1β (ab9722), MCP-1 (ab25124), VCAM-1(ab134047) and TGF-β1 (ab215715) were purchased from Abcam (Cambridge, United Kingdom); Mouse antibody against β-Actin (A5441) was from Sigma-Aldrich; Goat Serum Donor Herd (G6767) was from MilliporeSigma (Burlington, MA, USA); mouse IgG blocking reagent (MKB-2213) was from Vector Laboratories (Peterborough, United Kingdom); Secondary antibodies IRDye 800-conjugated affinity purified goat anti-rabbit IgG (92632211) was purchased from BioConcept (Alschwil, Switzerland) and Alexa fluor 680-conjugated goat anti-mouse IgG (A-21057) was from Invitrogen (Lucerne, Switzerland). Alexa Fluor 488-conjugated goat anti-rabbit IgG (H + L) secondary Ab (A-11008), Alexa Fluor 488-conjugated goat anti-mouse IgG (H + L) secondary Ab (A-11001), Alexa Fluor 568-conjugated goat anti-Mouse IgG (H + L) Highly Cross-Adsorbed Secondary Ab (A-11031), MitoSox (M36008), and Hoechst 33342 (62249) were from Thermo Fisher Scientific (Waltham, MA, USA). Written consent was obtained from the participants.

### Animals

*Arg-II*^−/−^ mice were kindly provided by Dr. William O’Brien^36^ and backcrossed to C57BL/6 J for more than 10 generations. Genotypes of mice were confirmed by polymerase chain reaction (PCR) as previously described^36^. Offspring of WT and *arg-II*^−/−^ mice were generated by interbred from hetero/hetero cross. Mice were housed at 23 °C with 12-h-light-dark cycle. Animals were fed a normal chow diet and have free access to water. Male and female mice at age of 7–8 months (young) or 24–28 months (old) were sacrificed and the left kidney was snap frozen in liquid nitrogen, kept at −80 °C until use. The right kidney was cut transversely, fixed with 3.7% paraformaldehyde, and then embedded in paraffin for immunofluorescence staining experiments. Experimental work with animals was approved by the Ethical Committee of Veterinary Office of Fribourg Switzerland (2013_08_FR and 2016_43_FR) and performed in compliance with guidelines on animal experimentation at our institution.

### Generation of recombinant adenoviral constructs

Recombinant adenoviral vectors expressing murine *arg-II* driven by the CMV promoter (rAd/CMV-*arg-II*) or shRNA targeting human *arg-II* driven by the U6 promoter (rAd/U6-*hArg-II*^shRNA^) were produced with the Gateway Technology (Invitrogen life Technologies) according to the manufacturer’s instruction. rAd/CMV-*LacZ* and rAd/U6-*LacZ*^shRNA^ served as the respective control for rAd/CMV-*arg-II* and rAd/U6-*hArg-II*^shRNA^. The pCMV6 construct encoding the murine *arg-II* was purchased from Origene. The human *arg-II* targeting sequence is indicated in bold as shown below (please note that only the sense strand is shown): CACC**GCGAGTGCATTCCATCCTGAA**CGAATTCAGGATGGAATGCACTCGC.

### Cell culture experiments and adenoviral gene transduction

The immortalized human renal proximal tubular cell line (HK-2) was purchased from ATCC and cultured at 37 °C in 5% CO_2_/95% air DMEM/Ham’s F-12 (1:1 v/v, Gibco) medium (Gibco) supplemented with 10% heat-inactivated fetal bovine serum (Gibco) and 1% penicillin/streptomycin. The media was changed every other day. For experiments, cells were seeded in six-well plates with a density of 2 × 10^5^ cells per well. After reaching 80% confluence, the cells were transduced either with rAd/U6-LacZshRNA as control or rAd/U6-*hArg-II*^shRNA^. The effect of *arg-II* silencing on TNFα-induced Arg-II protein levels and VCAM-1 and ICAM-1 levels were then investigated. To study the role of enhanced Arg-II on TGF-β1 production and mitochondrial ROS generation, the cells were transduced by rAd/CMV empty vector as control or rAd/CMV-h*Arg-II* for 24 h (50–100 MOI) and then cultured in complete medium for 2 days. Before experiments, the transduced cells were serum starved for 24 h. The cells were then harvested in a lysis buffer for immunoblotting of Arg-II, TGF-β1, and β-actin as described below in the section “immunoblotting”. In another series of experiments, analysis of mitochondrial oxidative stress was performed after the above transduction procedure as described below.

### Mitochondrial superoxide detection (MitoSox staining)

Mitochondrial superoxide generation was studied using MitoSox^11,37^. The cells were incubated with MitoSox at the concentration of 5 μmol/L for 10 min. After washing, the cells were then fixed with 3.7% of paraformaldehyde followed by counterstaining with Hoechst 33342 and then subjected to imaging under the Leica TCS SP5 confocal laser microscope with ×63 objectives. To study mitochondrial ROS generation, some cells were treated with rotenone (2 μmol/L, 1 h) followed by subjection to MitoSox as above described.

### Immunoblotting

Tissue and cell lysate preparation, SDS-PAGE and immunoblotting, antibody incubation, and signal detection were performed as described previously^11^. Ponceau staining and β-actin were used as the loading control. The fine powder was prepared from frozen kidney tissues using a mortar and pestle in a liquid nitrogen bath on dry ice. A portion of fine powder was then homogenized (XENOX-Motorhandstück MHX homogenizer) on ice in 150 µl of ice-cold lysis buffer with the following composition (mmol/L): Tris (20, pH 8.0, NaCl (138), KCl (2.7 × 10^−3^), MgCl (1 × 10^−6^), CaCl_2_ (1.0), NaVO_4_ (1.0), NaF (20), EDTA (5), glycerol (5%), NP-40 (1%), protease inhibitor cocktail (B14002 Biotool, Munich, Germany) and phosphatase inhibitor cocktail (B15002; Biotool, Munich, Germany). Homogenates were centrifuged (Sorvall Legend Micro 17R) at 13,800 × *g* for 15 min at 4 °C. Protein concentrations of the supernatant were then determined by the Lowry method (500-0116, Bio-Rad). Equal amount of protein from each sample was heated at 75 °C for 15 min in Laemmli buffer and separated by SDS-PAGE electrophoresis. Proteins in the SDS-PAGE gel were then transferred to PVDF membranes which were blocked with PBS-Tween-20 supplemented with 5% nonfat dry milk. The membranes were then incubated with the corresponding primary antibody overnight at 4 °C with gentle agitation. After washing with blocking buffer, the membranes were then incubated with corresponding anti-mouse (Alexa Fluor 680-conjugated) or anti-rabbit (IRDye 800-conjugated) secondary antibodies. Dilutions of each primary antibody were presented in Supplemental Table 1. Signals were visualized using the Odyssey Infrared Imaging System (LI-COR Biosciences, USA) and quantified by NIH Image J 1.60 (US NIH). All blots derive from the same experiments and were processed in parallel.

### Real-time quantitative RT-PCR

mRNA expression of the several inflammatory markers, *collagens, p16*^*INK4a*^*, arg-II, gapdh*, and Ribosomal Protein S12 (*rps12*) was measured by two-step quantitative Real Time-PCR as described previously^11^. Total RNA was extracted from kidney or HK-2 cells with Trizol Reagent (TR-118, Molecular Research Center, Inc., Cincinnati, OH, USA) following the manufacturer’s protocol. Real-time PCR reaction was performed with the GOTaq® qPCR Master Mix (A6001, Promega) and iCycler system (Bio-Rad). The mRNA expression levels of all genes were quantified using the standard curve method and were further normalized to the reference gene *rps12* or *gapdh*.

The following primer sequences of mouse origin were used:

*collagen 1α1*-F: 5′-TGG CCA AGA AGA CAT CCC TGA AGT C-3′

*collagen 1α1*-R: 5′-GGC AGA TAC AGA TCA AGC ATA CCT CGG-3′

*collagen 1α2*-F: 5′-CTG GTC TTA CTG GGA ACT TTG CTG C-3′

*collagen 1α2*-R: 5′-CCA ACA GCA CCA GGA GGG CC-3′

*collagen 3α1*-F: 5′-CAA ACA CGC AAG GCA ATG AGA CTA CC-3′

*collagen 3α1*-R: 5′-AGG GCC AAT GTC CAC ACC AAA TTC-3′

*f4/80-*F: 5′-TGG CTG CCT CCC TGA CTT TC-3′

*f4/80-*R: 5′-CAA GAT CCC TGC CCT GCA CT-3′

*icam-1*-F: 5′-TGC TTT GAG AAC TGT GGC AC-3′

*icam-1*-R: 5′-GCT CAG TAT CTC CTC CCC AC-3′

*il6*-F: 5′-GAC AAC CAC GGC CTT CCC TA-3′

*il6*-R: 5′-GCC TCC GAC TTG TGA AGT GGT-3′

*il-1β*-F: 5′-GCA ACT GTT CCT GAA CTC AAC T-3′

*il-1β*-R: 5′-TCT TTT GGG GTC CGT CAA CT-3′

*inos*-F: 5′-GGC AAA CCC AAG GTC TAC GTT-3′

*inos*-R: 5′-TCG CTC AAG TTC AGC TTG GT-3′

*mcp1*-F: 5′-AGC ACC AGC CAA CTC TCA C-3′

*mcp1*-R: 5′-TCT GGA CCC ATT CCT TCT TG-3′

*p16*^*INK4a*^-F: GAA CTC TTT CGG TCG TAC

*p16*^*INK4a*^-R: GCA GAA GAG CTG CTA CGT

*rps12*-F: 5′-GAA GCT GCC AAA GCC TTA GA-3′

*rps12*-R: 5′-AAC TGC AAC CAA CCA CCT TC-3′

*tnf-α*-F: 5′-GGC AGG TCT ACT TTG GAG TCA TTG C-3′

*tnf-α*-R: 5′-ACA TTC GAG GCT CCA GTG AAT TCG G-3′

*vcam-1*-F: 5′-ACA GAC AGT CCC CTC AAT GG-3′

*vcam-1*-R: 5′-ACA GTG ACA GGT CTC CCA TG-3′

*tgf-β1*-F: 5′-TGG AGC AAC ATG TGG AAC TC-3′

*tgf-β1*-R: 5′-CAG CAG CCG GTT ACC AAG-3′

The primer sequences of human are as follows:

*arg-II*-F: 5′-GGCTGAGGTGGTTAGCAGAG-3′

*arg-II*-R: 5′-CTGGCTGTCCATGGAGATTT-3′

*gapdh*-F: 5′-TGCACCACCAACTGCTTAGC-3′

*gapdh*-R: 5′-GGCATGGACTGTGGTCATGAG-3′

### Immunofluorescence staining

Kidneys were isolated and fixed with 3.7% paraformaldehyde and embedded in paraffin. Horizontal central transverse sections through the middle of the kidney (5 μm) were prepared with Microtome. After deparaffinization in xylene (3 times, 5 min for each), the sections were treated in ethanol (twice in 100% ethanol, twice in 95% ethanol, and once in 80%, 75%, 50% ethanol for 5 min, sequentially) followed by antigen retrieval (EDTA buffer, pH 8.0 for Arg-II, IL-1β, MCP-1, VCAM-1, ACE1, PCNA, and TGF-β1; Tris- EDTA buffer, pH 9.0 for F4/80 and pT389-S6K1; citrate buffer, pH 6.0 for p16^INK4a^) in a pressure cooker. For immunofluorescence staining of Arg-II, IL-1β, MCP-1, VCAM-1, F4/80, p-T389-S6K1, and p16^INK4α^, the transverse sections (5 μm) were blocked with 1% BSA and 10% goat serum for 1 h and incubated with primary antibodies at 4 °C overnight and subsequently with Alexa Fluor 488–conjugated goat anti-rabbit IgG (H + L) or goat anti-mouse IgG (H + L) for 2 h at room temperature in darkness followed by counterstaining with 300 nmol/L DAPI for 3 min. Negative controls were performed by omitting the primary antibodies.

For co-immunofluorescence staining of VCAM-1/ACE1, PCNA/ACE1, TGF-β1/ACE1, IL-1β/ACE1, MCP-1/ACE1, primary antibodies of different species are used: Transverse sections (5 μm) were blocked with mouse IgG blocking reagent for 3 h and then with PBS that contained 1% BSA and 10% goat serum for 1 h. Then sections were incubated with rabbit anti-VCAM-1, PCNA, TGF-β1, IL-1β, and MCP-1, with mouse-anti ACE-1 antibody, respectively, at 4 °C overnight and subsequently with Alexa Fluor 488–conjugated goat anti-rabbit IgG (H + L) and Alexa Fluor 568-conjugated goat anti-Mouse IgG (H + L) Highly Cross-Adsorbed Secondary Antibody for 2 h at room temperature, followed by counterstaining with 300 nmol/L DAPI for 3 min. Immunofluorescence signals were visualized under Leica DM6B Navigator.

The intensity of the fluorescence was quantified by densitometric analysis of the target proteins by Leica Application Suite X (LAS X) software. The background signal was determined in non-stained areas in each section. The threshold was adjusted according to the background signal. Positive signals were outlined manually according to their signal. Within these defined areas, the mean value of signal intensities was determined using the “intensity” function in LAS X software. Data are expressed as mean values per mm^2^.

### Masson’s trichrome staining

Central transverse sections (5 µm) were subjected to Masson’s trichrome (Abcam, ab150686, Cambridge, United Kingdom) staining according to the manufacturer’s instructions. Collagen deposition was determined on the trichrome-stained kidney sections. The positive area was quantitatively measured using NIH Image J 1.60 software as described previously^38,39^. For Glomerulosclerosis (GS, fibrotic glomeruli) score, it was done as follows: 0, no fibrosis; 1, fibrosis area <25%; 2, fibrosis area = 26–50%; 3, fibrosis area = 51–75%; and 4, fibrosis area >75% as described previously^40,41^. The Glomerulosclerosis score was defined by counted of fibrotic glomeruli in microscopic fields until a total of 30 glomeruli had been counted.

### TUNEL assay

After deparaffinization and rehydration, transverse sections (5 μm) were pretreated with Citrate buffer (0.1 M, pH 6.0). The detection of apoptotic cells in the kidney was then carried out with the “In Situ Cell Death Detection Kit, TMR red” (TUNEL) (Roche Applied Science, #12156792910, Basel, Switzerland) according to the manufacturer’s instruction. The signals were visualized under Leica DM6B Navigator. Apoptotic cells were quantified and presented as a number of TUNEL positive cells/mm^2^.

### Statistics

Data are given as mean ± SEM. In all experiments, *n* represents the number of animals. After determination of Gaussian distributions using the Kolmogorov-Smirnov test, statistical analysis for normally distributed values was performed with Student’s unpaired *t*-test or analysis of variance (ANOVA) with Bonferroni post hoc test, or, if non-normally distributed values, Mann–Whitney test or the Kruskal–Wallis test was used. Differences in mean values were considered significant at two-tailed *P* ≤ 0.05.

## Supplementary information

Supplementary Information

nr-reporting-summary

## Data Availability

All the data supporting the findings are available from the authors upon request to any qualified researchers

## References

[1] O’Sullivan ED, Hughes J, Ferenbach DA (2017). Renal aging: causes and consequences. J. Am. Soc. Nephrol..

[2] Schmitt R, Melk A (2017). Molecular mechanisms of renal aging. Kidney Int..

[3] Hommos MS, Glassock RJ, Rule AD (2017). Structural and functional changes in human kidneys with healthy aging. J. Am. Soc. Nephrol..

[4] European-Commission. The 2015 ageing report. *Eur. Econ.* https://ec.europa.eu/economy_finance/publications/european_economy/2015/pdf/ee3_en.pdf (2015)

[5] Carrero JJ, Hecking M, Chesnaye NC, Jager KJ (2018). Sex and gender disparities in the epidemiology and outcomes of chronic kidney disease. Nat. Rev. Nephrol..

[6] Bikbov B, Perico N, Remuzzi G, on behalf of the, G. B. D. G. D. E. G. (2018). Disparities in chronic kidney disease prevalence among males and females in 195 countries: analysis of the global burden of disease 2016 study. Nephron.

[7] Yepuri G (2012). Positive crosstalk between arginase-II and S6K1 in vascular endothelial inflammation and aging. Aging Cell.

[8] Xiong, Y., Yepuri, G., Montani, J.-P., Ming, X.-F. & Yang, Z. Arginase-II deficiency extends lifespan in mice. *Front. Physiol*. 10.3389/fphys.2017.00682 (2017). 10.3389/fphys.2017.00682 PMC5596098 28943853

[9] Xiong Y (2017). Arginase-II promotes tumor necrosis factor-alpha release from pancreatic acinar cells causing beta-cell apoptosis in aging. Diabetes.

[10] Yang, Z. & Ming, X. F. Arginase: the emerging therapeutic target for vascular oxidative stress and inflammation. *Front Immunol*. 10.3389/fimmu.2013.00149 (2013). 10.3389/fimmu.2013.00149 PMC3679468 23781221

[11] Ming, X. F. et al. Arginase II promotes macrophage inflammatory responses through mitochondrial reactive oxygen species, contributing to insulin resistance and atherogenesis. *J. Am. Heart Assoc.*10.1161/JAHA.112.000992 (2012). 10.1161/JAHA.112.000992 PMC3487353 23130157

[12] Multhaupt H, Fritz P, Schumacher K (1987). Immunohistochemical localisation of arginase in human liver using monoclonal antibodies against human liver arginase. Histochemistry.

[13] Sekine S, Ogawa R, McManus MT, Kanai Y, Hebrok M (2009). Dicer is required for proper liver zonation. J. Pathol..

[14] Levillain O, Balvay S, Peyrol S (2005). Localization and differential expression of arginase II in the kidney of male and female mice. Pflug. Arch..

[15] Huang, J. et al. Genetic targeting of Arginase-II in mouse prevents renal oxidative stress and inflammation in diet-induced obesity. *Front. Physiol*. 10.3389/fphys.2016.00560 (2016). 10.3389/fphys.2016.00560 PMC5118905 27920727

[16] Vio, C. P, et al. Imbalance in renal vasoactive enzymes induced by mild hypoxia: angiotensin-converting enzyme increases while neutral endopeptidase decreases. *Front. Physiol*. 10.3389/fphys.2018.01791 (2018). 10.3389/fphys.2018.01791 PMC6297360 30618804

[17] Levillain O, Diaz JJ, Blanchard O, Dechaud H (2005). Testosterone down-regulates ornithine aminotransferase gene and up-regulates arginase II and ornithine decarboxylase genes for polyamines synthesis in the murine kidney. Endocrinology.

[18] Hayashi T (2006). Modulating role of estradiol on arginase II expression in hyperlipidemic rabbits as an atheroprotective mechanism. Proc. Nalt Acad. Sci. USA.

[19] Traish AM (2003). Sex steroid hormones differentially regulate nitric oxide synthase and arginase activities in the proximal and distal rabbit vagina. Int. J. Impot. Res..

[20] Liu, C. et al. Targeting arginase-II protects mice from high-fat-diet-induced hepatic steatosis through suppression of macrophage inflammation. *Sci. Rep*. 10.1038/srep20405 (2016). 10.1038/srep20405 PMC4742779 26846206

[21] Saxton RA, Sabatini DM (2017). mTOR signaling in growth, metabolism, and disease. Cell.

[22] Herranz D, Serrano M (2010). SIRT1: recent lessons from mouse models. Nat. Rev. Cancer.

[23] Nitta K, Okada K, Yanai M, Takahashi S (2013). Aging and chronic kidney disease. Kidney Blood Press. Res..

[24] Yang HC, Fogo AB (2014). Fibrosis and renal aging. Kidney Int. Suppl..

[25] Caldwell RB, Toque HA, Narayanan SP, Caldwell RW (2015). Arginase: an old enzyme with new tricks. Trends Pharm. Sci..

[26] Raup-Konsavage WM (2017). Arginase-2 mediates renal ischemia-reperfusion injury. Am. J. Physiol. Ren. Physiol..

[27] Zhang X (2006). TIMP-1 promotes age-related renal fibrosis through upregulating ICAM-1 in human TIMP-1 transgenic mice. J. Gerontol. A Biol. Sci. Med Sci..

[28] Rodwell, G. E. et al. A transcriptional profile of aging in the human kidney. *PLoS Biol*. 10.1371/journal.pbio.0020427 (2004). 10.1371/journal.pbio.0020427 PMC532391 15562319

[29] Ma TT, Meng XM (2019). TGF-beta/Smad and renal fibrosis. Adv. Exp. Med. Biol..

[30] Guzzi, F., Cirillo, L., Roperto, R. M., Romagnani, P. & Lazzeri, E. Molecular mechanisms of the acute kidney injury to chronic kidney disease transition: an updated view. *Int. J. Mol. Sci*. 10.3390/ijms20194941 (2019). 10.3390/ijms20194941 PMC6801733 31590461

[31] Lazzeri E (2018). Endocycle-related tubular cell hypertrophy and progenitor proliferation recover renal function after acute kidney injury. Nat. Commun..

[32] Ansermet, C. et al. Renal tubular arginase-2 participates in the formation of the corticomedullary urea gradient and attenuates kidney damage in ischemia-reperfusion injury in mice. *Acta Physiol*. 10.1111/apha.13457 (2020). 10.1111/apha.13457 32072766

[33] Hara M (2020). Arginase 2 is a mediator of ischemia–reperfusion injury in the kidney through regulation of nitrosative stress. Kidney Int..

[34] Grgic I (2012). Targeted proximal tubule injury triggers interstitial fibrosis and glomerulosclerosis. Kidney Int..

[35] Liu BC, Tang TT, Lv LL, Lan HY (2018). Renal tubule injury: a driving force toward chronic kidney disease. Kidney Int..

[36] Shi O, Morris SM, Zoghbi H, Porter CW, O’Brien WE (2001). Generation of a mouse model for arginase II deficiency by targeted disruption of the arginase II gene. Mol. Cell Biol..

[37] Chavan, H. et al. Arsenite effects on mitochondrial bioenergetics in human and mouse primary hepatocytes follow a nonlinear dose response. *Oxid. Med. Cell Longev*. 10.1155/2017/9251303 (2017). 10.1155/2017/9251303 PMC5253485 28163822

[38] Landini G, Martinelli G, Piccinini F (2020). Colour Deconvolution - stain unmixing in histological imaging. Bioinformatics.

[39] Chen Y, Yu Q, Xu C-B (2017). A convenient method for quantifying collagen fibers in atherosclerotic lesions by ImageJ software. Int. J. Clin. Exp. Med..

[40] Guerrot, D. et al. Identification of periostin as a critical marker of progression/reversal of hypertensive nephropathy. *PLoS ONE*10.1371/journal.pone.0031974 (2012). 10.1371/journal.pone.0031974 PMC3293874 22403621

[41] Guerrot D (2011). Discoidin domain receptor 1 is a major mediator of inflammation and fibrosis in obstructive nephropathy. Am. J. Pathol..

