# Supplementary material for: Role of tubular epithelial arginase-II in renal inflammaging
Source: NPJ Aging Mech Dis. 2021 Mar 2;7:5. doi: 10.1038/s41514-021-00057-8 (PMC7925687; doi:10.1038/s41514-021-00057-8)
Supplement: Supplementary file 1 — Supplementary Information [file 41514_2021_57_MOESM1_ESM.pdf]

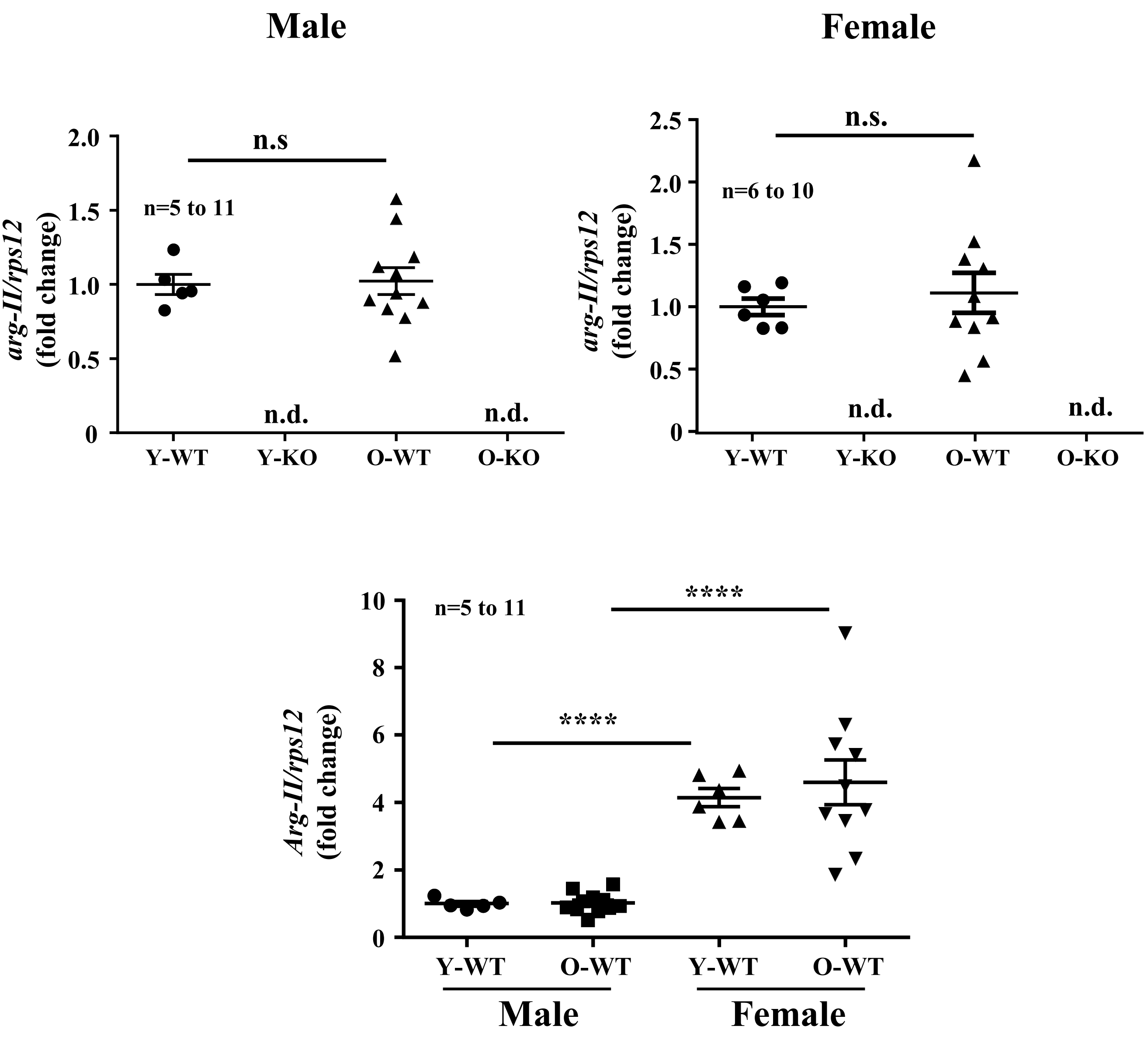

Supplementary Figure 1: mRNA levels of *arg-II* in kidneys of young and old male and female mice. mRNA expression levels of *arg-II* in kidneys were analyzed by qRT-PCR. *rps12* served as the reference. Y-WT, young WT; Y-KO, young *arg-II*$^{-/-}$; O-WT, old WT; O-KO, old *arg-II*$^{-/-}$. Data are expressed as the fold change to the Y-WT group. n indicates the number of animals used in the experimental groups. n.s. means not significant; ****P ≤ 0.0001 between the indicated groups.

Male

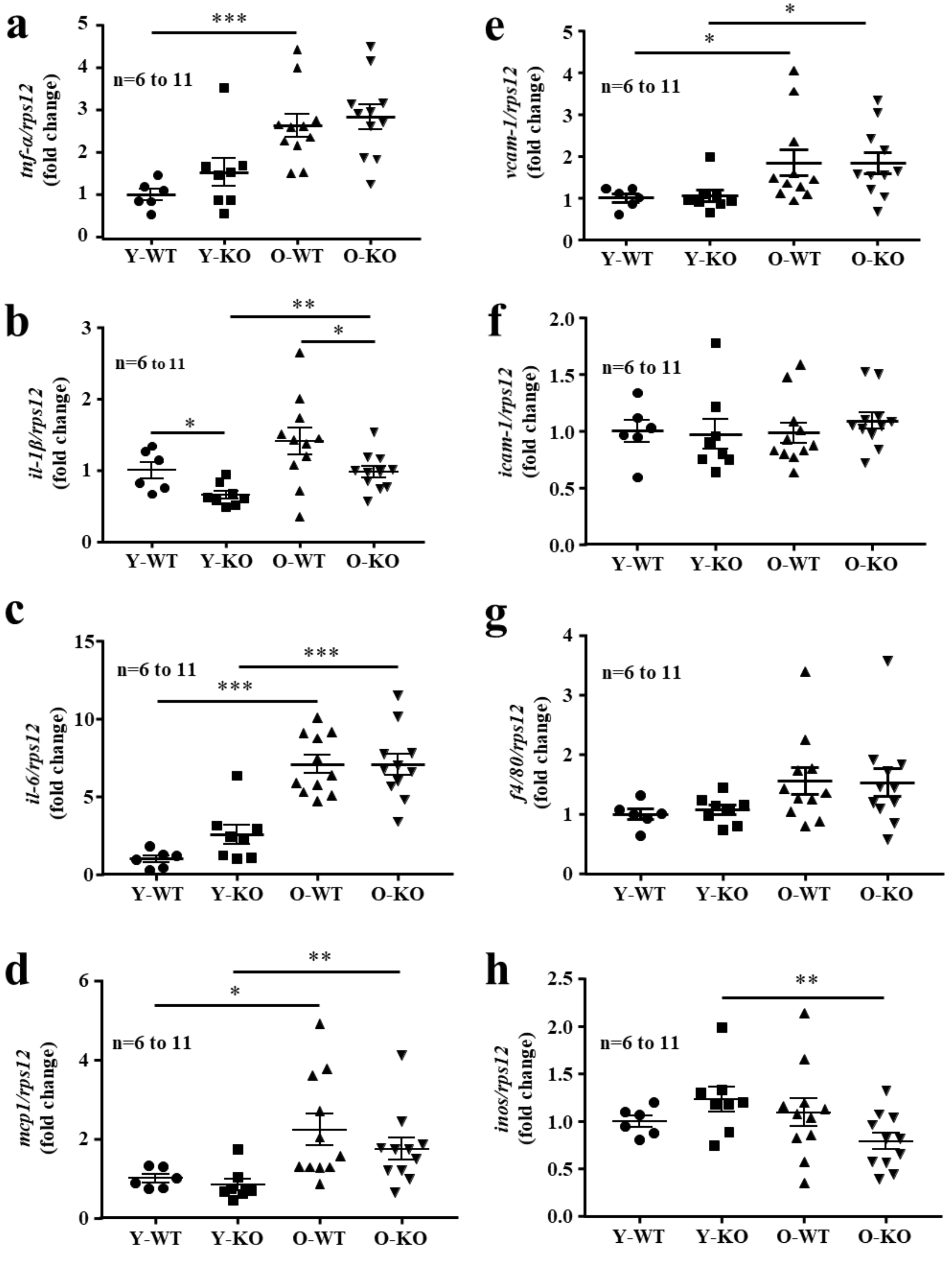

Supplementary Figure 2. Age-associated inflammation in males. (**a-h**) mRNA expression levels of *tnf-α, il-1β, il-6, mcp1,vcam-1, icam-1, f4/80* and *inos* in kidney were analyzed by qRT-PCR in male mice. *rps12* served as the reference. Y-WT, young WT; Y-KO, young *arg-II*$^{-/-}$; O-WT, old WT; O-KO, old *arg-II*$^{-/-}$. Data are expressed as the fold change to the Y-WT group. n indicates the number of animals used in the experimental groups. *P ≤ 0.05, **P ≤ 0.01, ***P ≤ 0.001.

Male

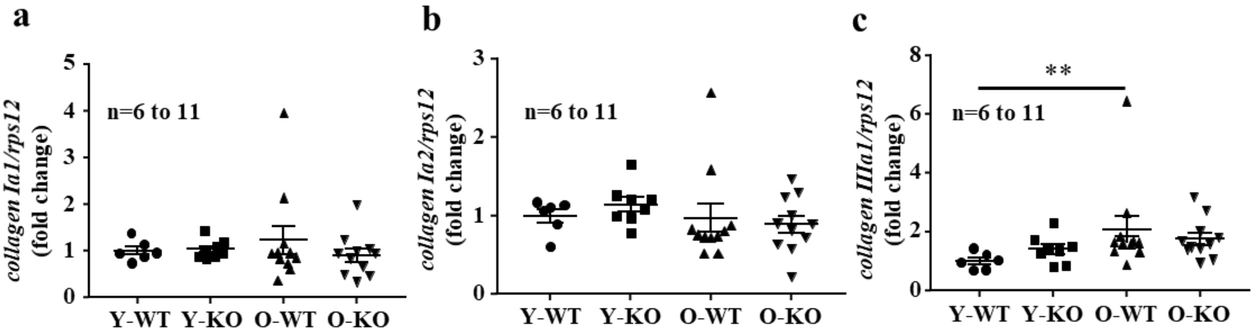

Supplementary Figure 3. Expression of *collagen Iα1, Iα2 and IIIα1* in males. mRNA expression levels of *collagen type Iα1* **(a)**, *Iα2* **(b)** and *IIIα1* **(c)** were analyzed by qRT-PCR in male mice, respectively. *rps12* was used as reference. Y-WT, young WT; Y-KO, young *arg-II*$^{-/-}$; O-WT, old WT; O-KO, old *arg-II*$^{-/-}$. Data are expressed as the fold change to the Y-WT group. n indicates the number of animals used in the experimental groups. **P ≤ 0.01.

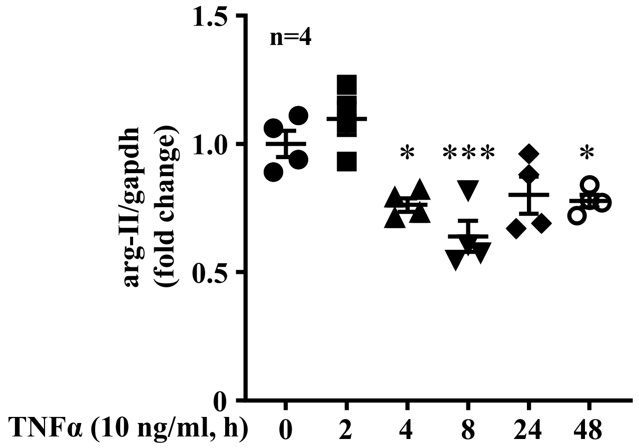

Suppl. Fig. 4. Effects of TNF-α on *arg-II* mRNA levels in HK-2 cells. HK-2 cells were serum starved for 24 hours, then incubated in the presence of TNF-α (10 ng/ml) for 0 to 48 hours. mRNA levels of *arg-II* were analyzed by qRT-PCR. *gapdh* was used as reference. Data are expressed as the fold change to control group. n indicates the number of animals used in the experimental groups. *P ≤ 0.05, ***P ≤ 0.001.

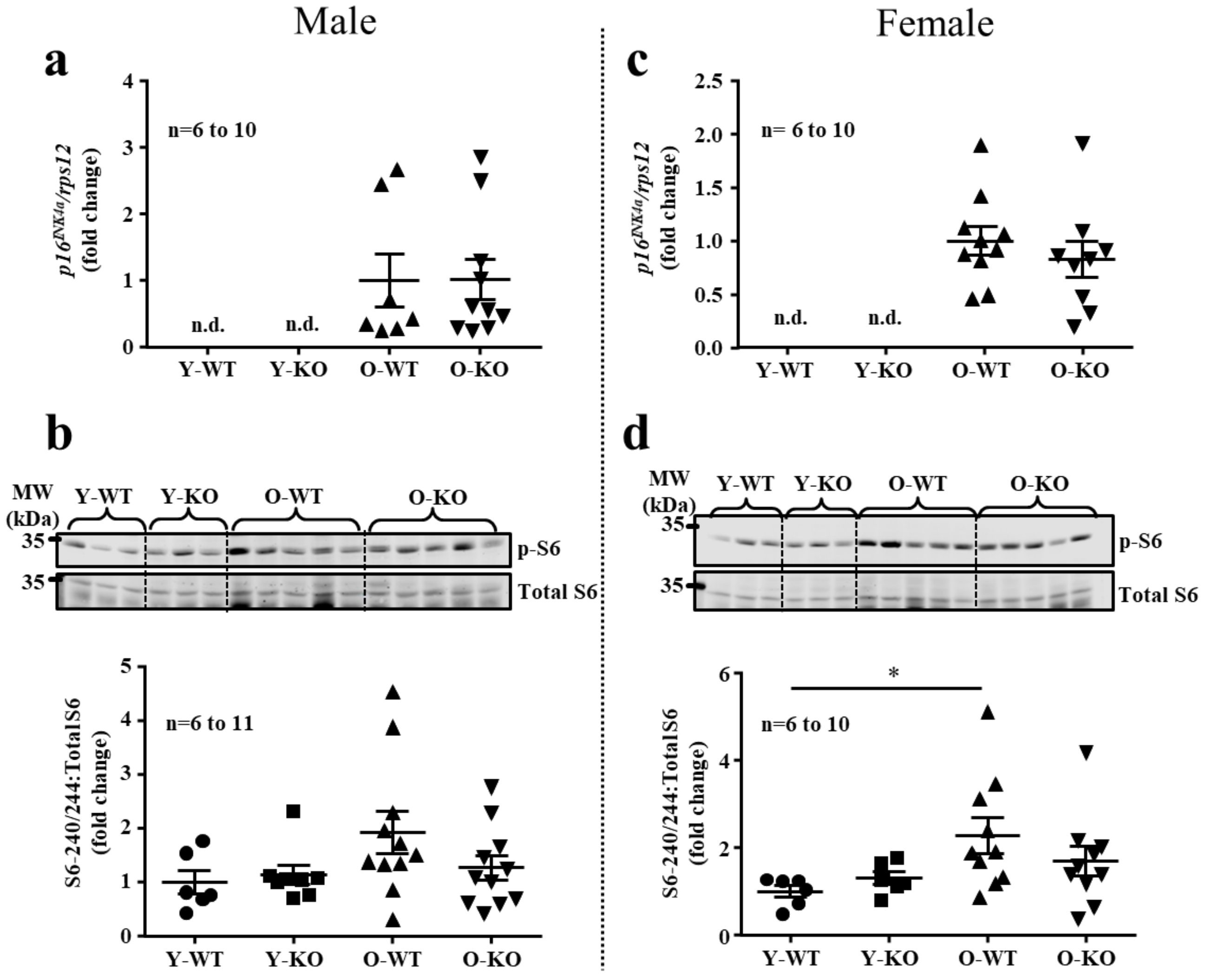

Supplementary Figure 5. ***Arg-II*** **deficiency does not have impact on** ***p16^{INK4a}*** **mRNA and p-S6 levels.** mRNA expression of *p16^{INK4a}* was analyzed by qRT-PCR in kidney tissue of male and female mice (**a**, **c**). Since *p16^{INK4a}* expression is not detectable in young mice, O-WT group was used as reference for fold change. *rps12* serves as reference gene. Immunoblotting analyses of phosphorylated ribosomal S6 protein (S6-S240/244) and total S6 in whole kidney lysates of male **(b)** and female **(d)** WT and *arg-II$^{-/-}$* mice. Y-WT, young wild type; Y-KO, young *arg-II$^{-/-}$*; O-WT, old wild type; O-KO, old *arg-II$^{-/-}$*. n indicates the number of animals in the experimental groups. *$p \leq 0.05$.

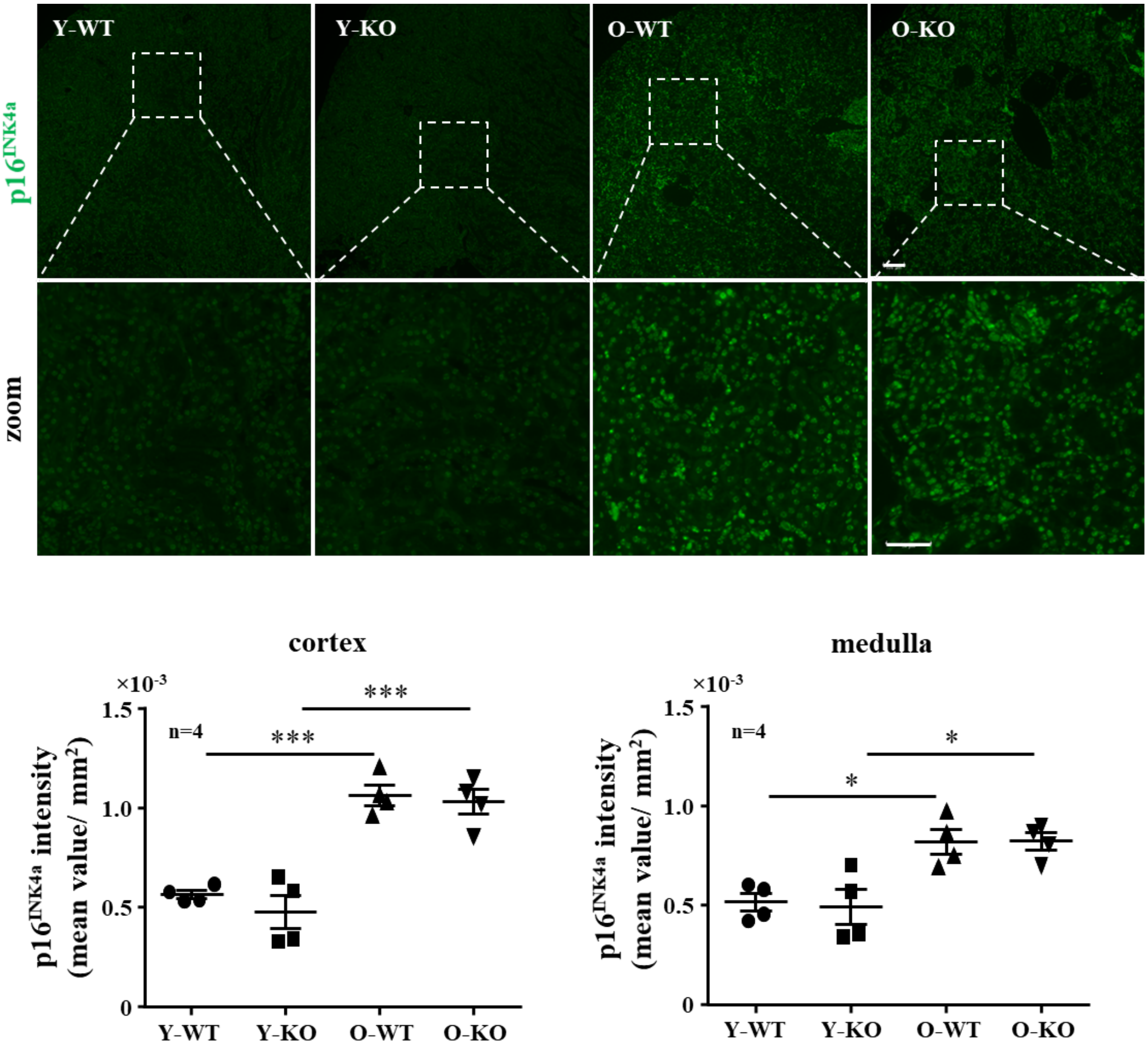

Supplementary Figure 6. *Arg-II* deficiency does not have impact on p16^{INK4a}. Central transverse renal sections (one section from each animal) were prepared from young and old WT and *arg-II*$^{-/-}$ female mice and subjected to immunofluorescence staining of p16^{INK4a}. Scale bar =50 µm in the upper panel and 20 µm in the lower panels, respectively. Y-WT, young WT; Y-KO, young *arg-II*$^{-/-}$; O-WT, old WT; O-KO, old *arg-II*$^{-/-}$. n indicates the number of animals used in the experimental groups. *P ≤ 0.05, ***P ≤ 0.001.

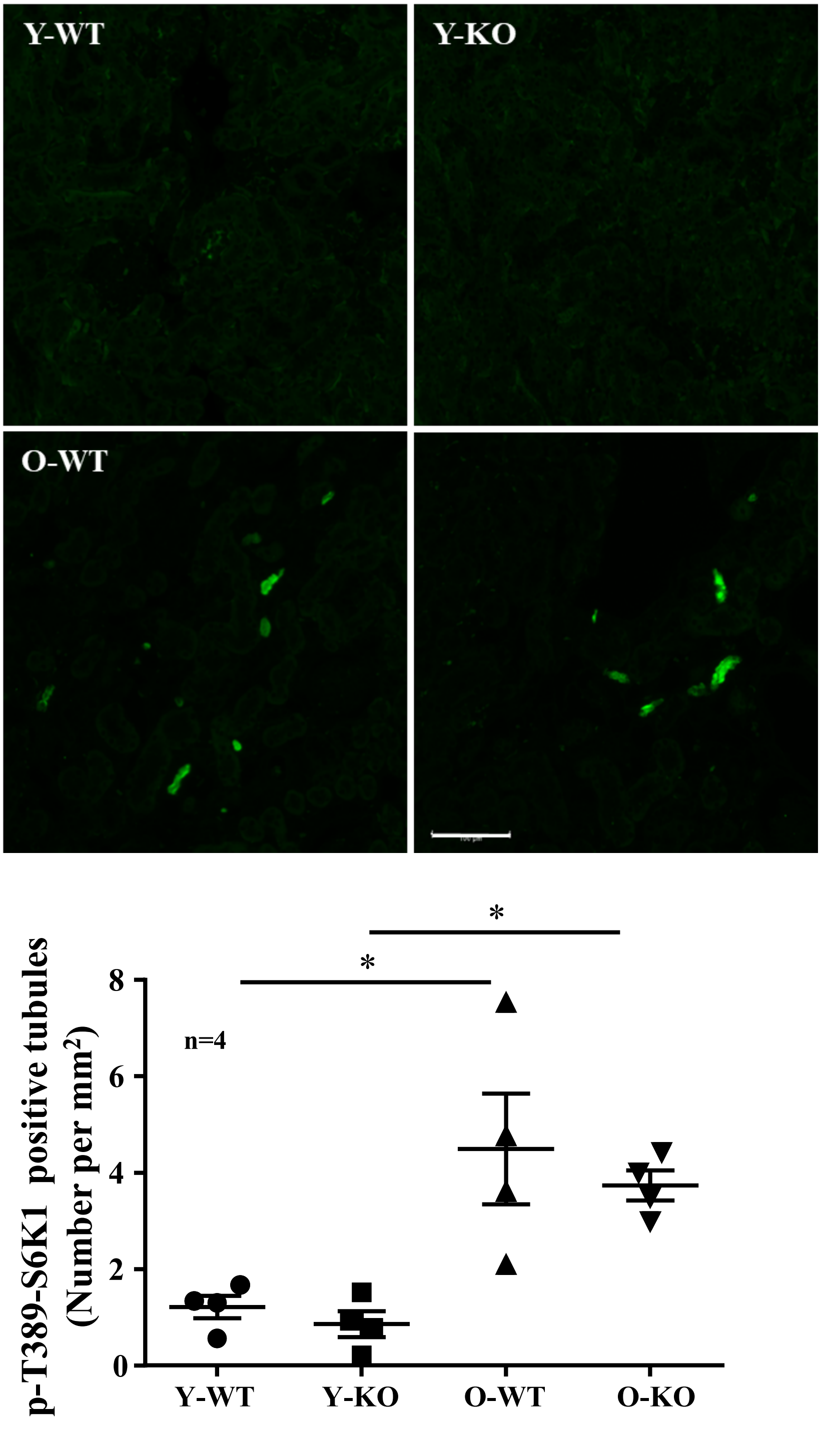

Supplementary Figure 7. *Arg-II* deficiency does not affect p-S6K (Thr389). Central transverse renal sections (one section from each animal) were prepared from young and old WT and *arg-II*$^{-/-}$ female mice and subjected to immunofluorescence staining of p-Thr389-S6K1. Scale bar = 100 µm. Y-WT, young WT; Y-KO, young *arg-II*$^{-/-}$; O-WT, old WT; O-KO, old *arg-II*$^{-/-}$. n indicates the number of animals used in the experimental groups. *P ≤ 0.05.

Supplementary Table 1. Antibody dilutions used for immunoblotting and immunofluorescence

Antibody target	Dilution
ACE1	IF 1:50
Arg-II	WB 1:800, IF 1:400
β-actin	WB 1:5,000
ICAM-1	WB 1:200
IL-1β	IF 1:200
F4/80	IF 1:200
MCP-1	IF 1:200
P16	IF 1:50
phospho S6-S240/244	WB 1:1,000
PCNA	IF 1:800
Phospho-p70 S6 Kinase-Thr389	IF 1:300
S6	WB 1:1,000
TGF-β1	WB 1:1,000, IF 1:500
VCAM-1	WB 1:1,000, IF 1:500
IRDye 800-conjugated affinity purified goat anti-rabbit IgG	WB 1:5,000
Alexa fluor 680-conjugated goat anti-mouse IgG	WB 1:5,000
Alexa Fluor 488-conjugated goat anti-rabbit IgG (H+L) secondary Ab	IF 1:400
Alexa Fluor 488-conjugated goat anti-mouse IgG (H+L) secondary Ab	IF 1:400
Alexa Fluor 568-conjugated goat anti-Mouse IgG (H+L) Highly Cross-Adsorbed Secondary Ab	IF 1:400

Figure.1 un-cropped images

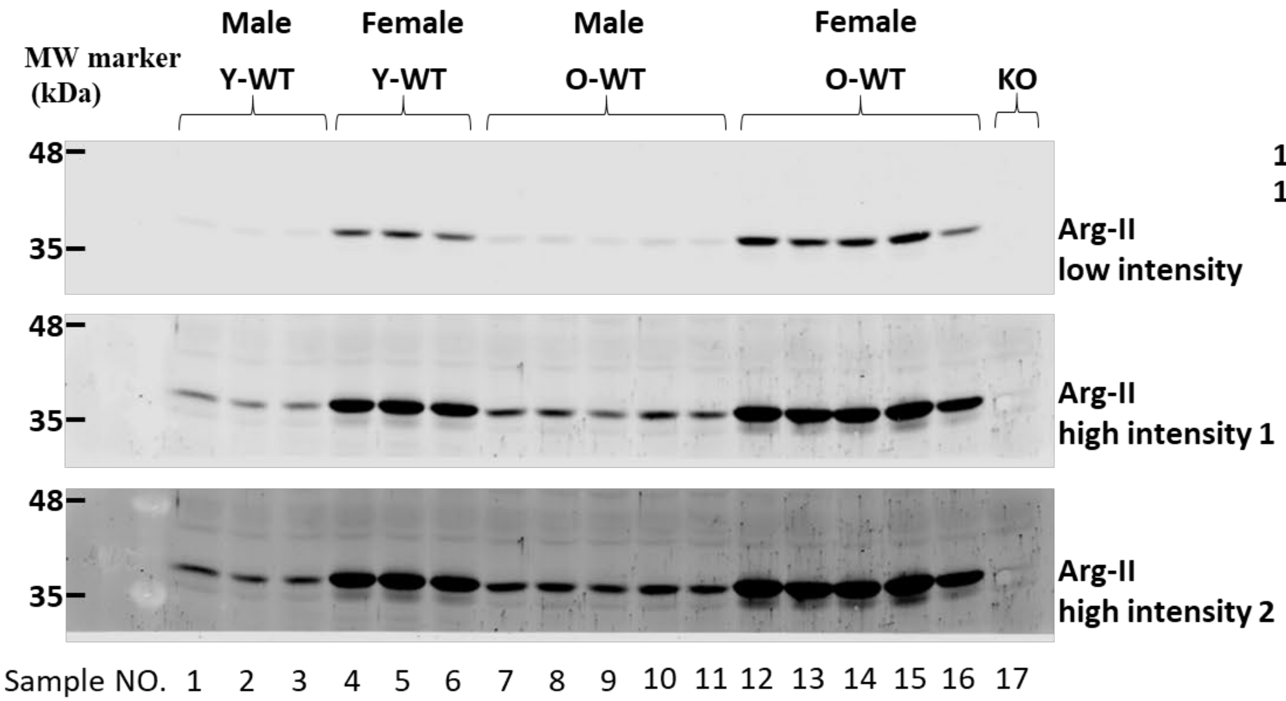

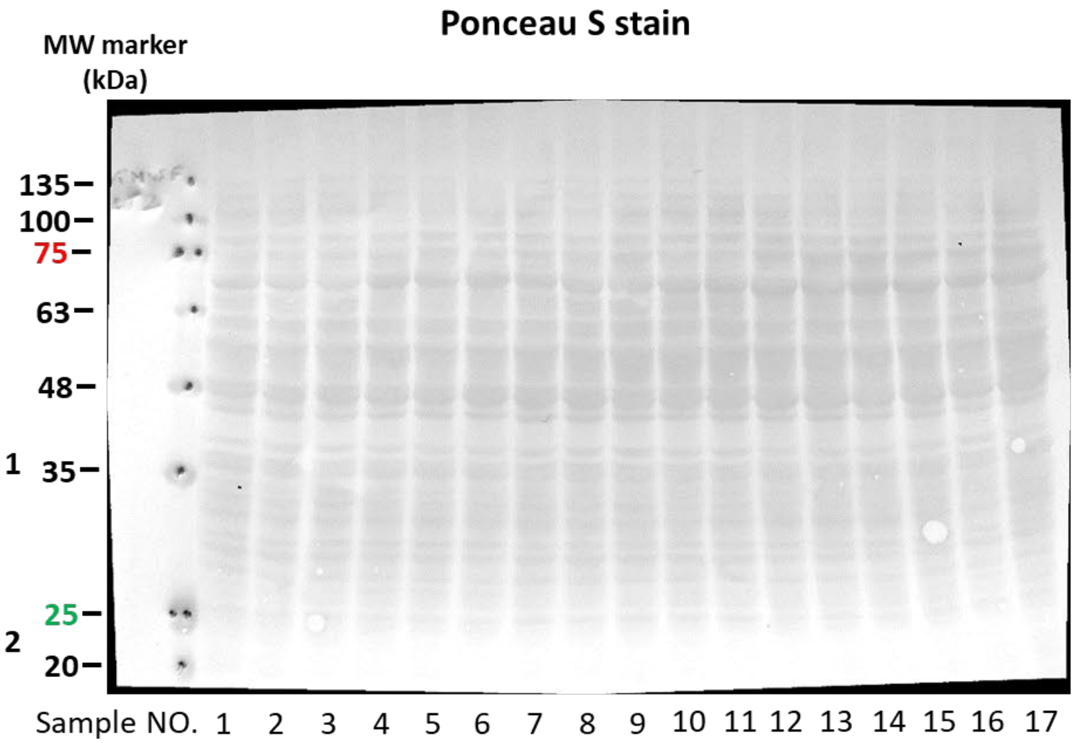

Figure.8a left panel un-cropped images

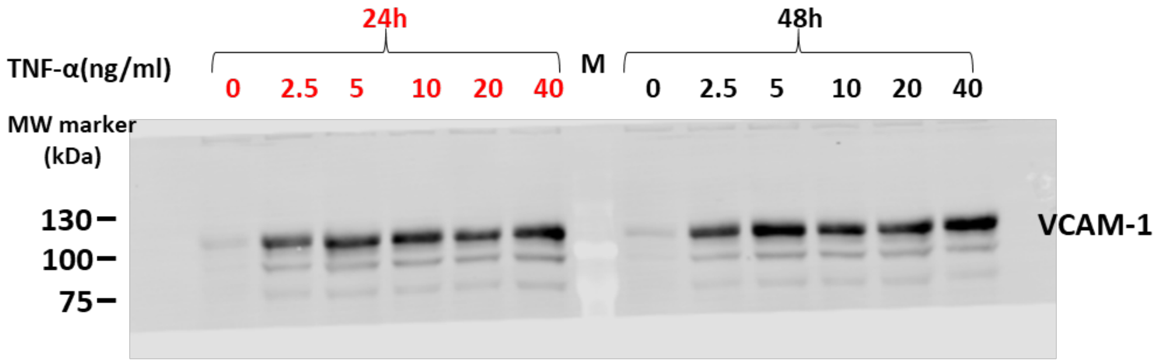

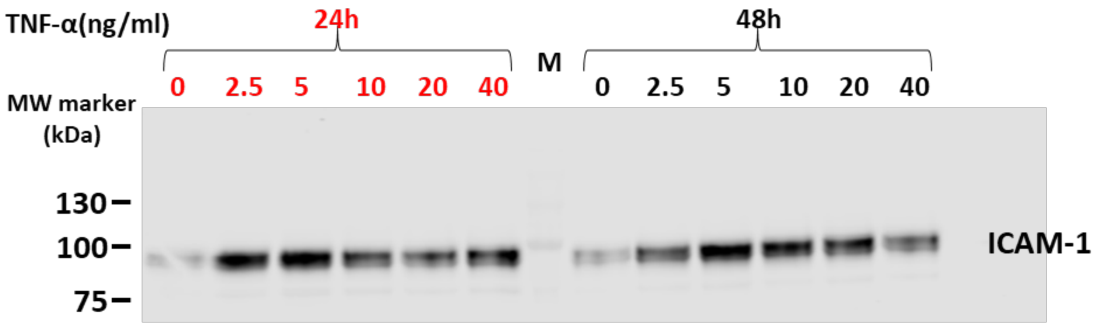

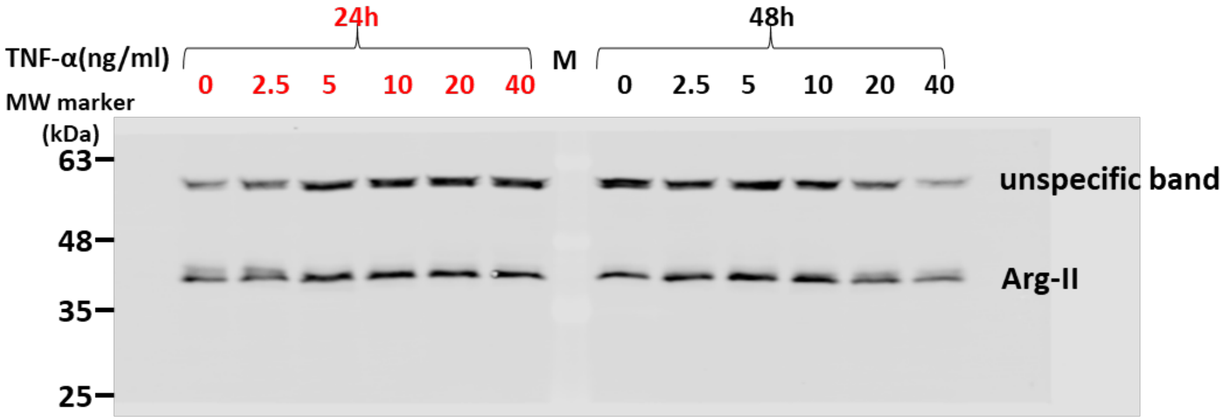

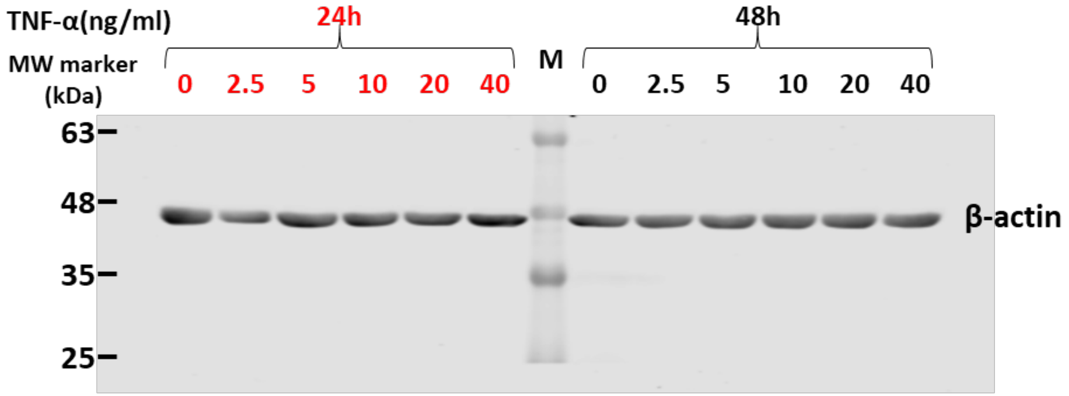

M/MW marker: molecule weight marker

Figure.8a right panel un-cropped images

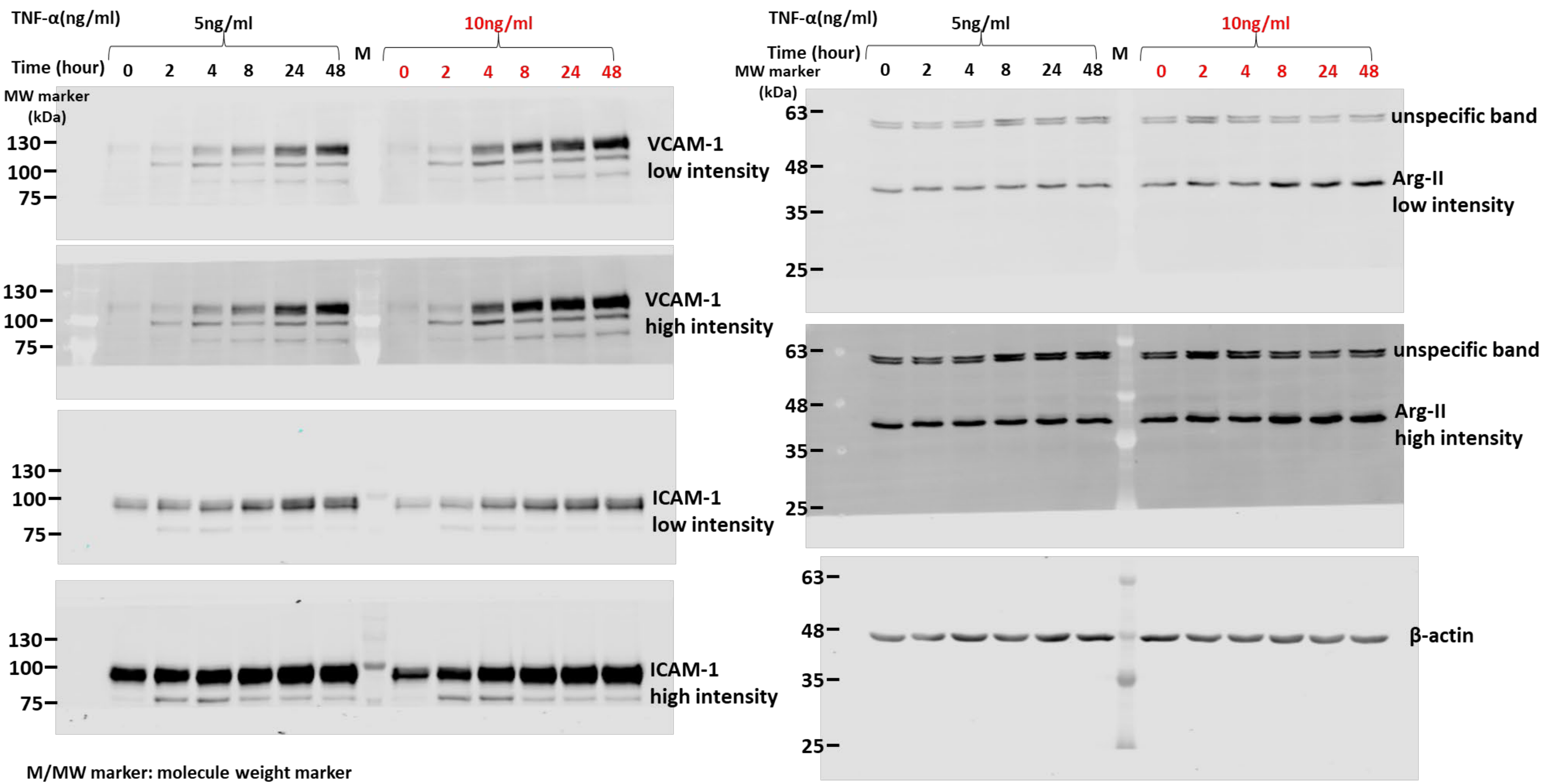

M/MW marker: molecule weight marker

Figure.8b un-cropped images

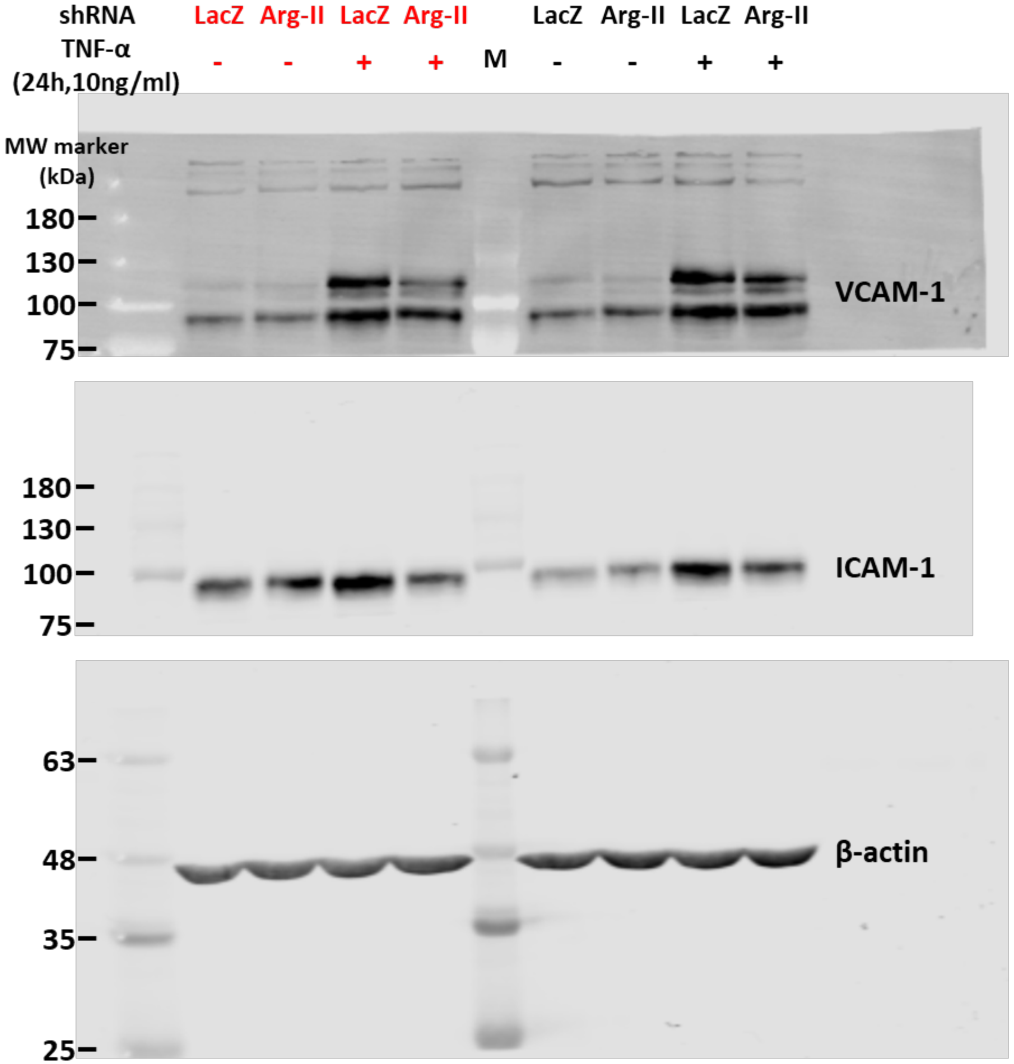

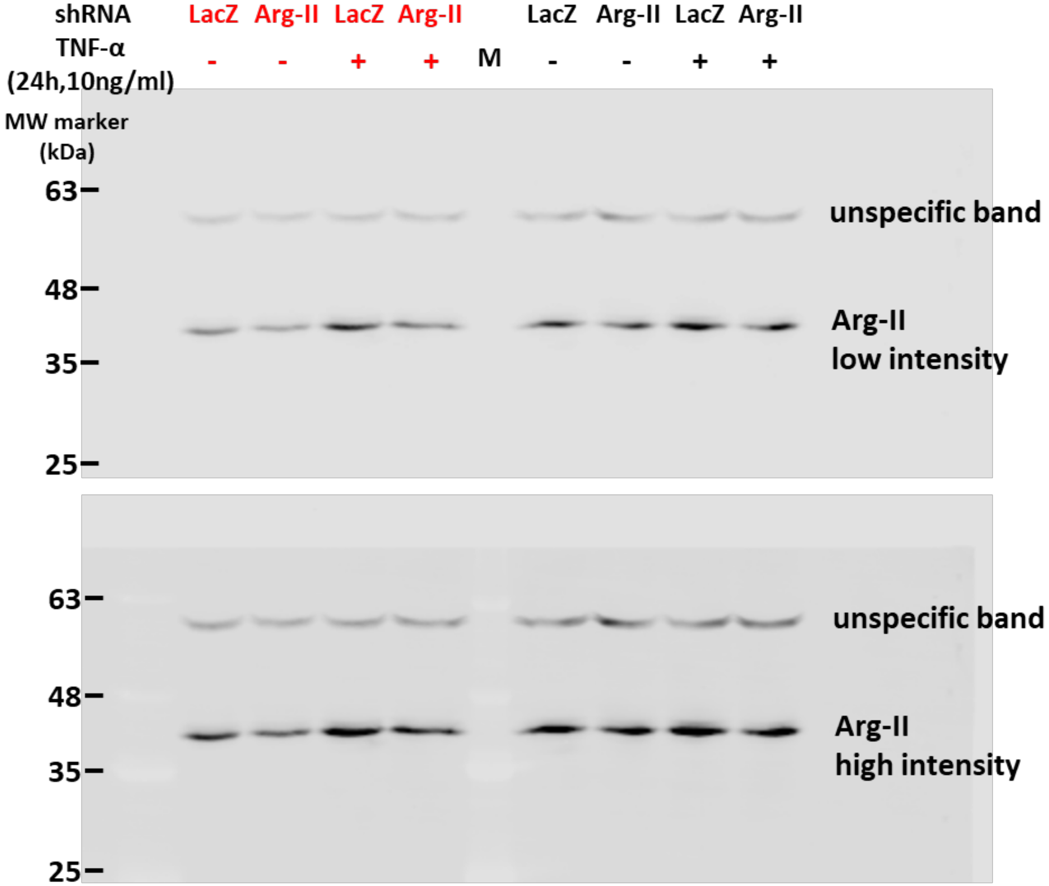

M/MW marker: molecule weight marker

Figure.9a un-cropped images

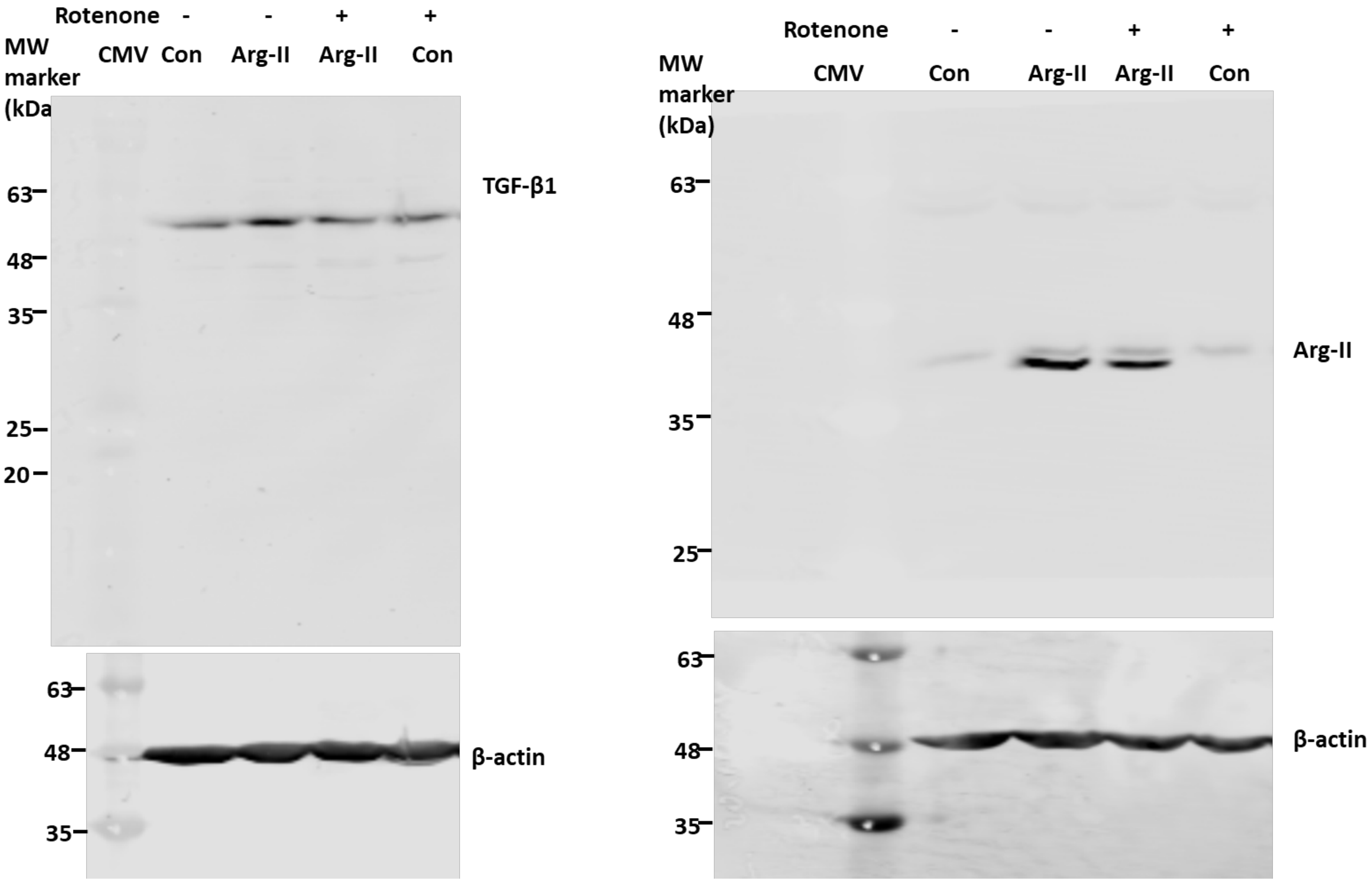

Suppl Fig.5 un-cropped images

Suppl Fig.5b Male

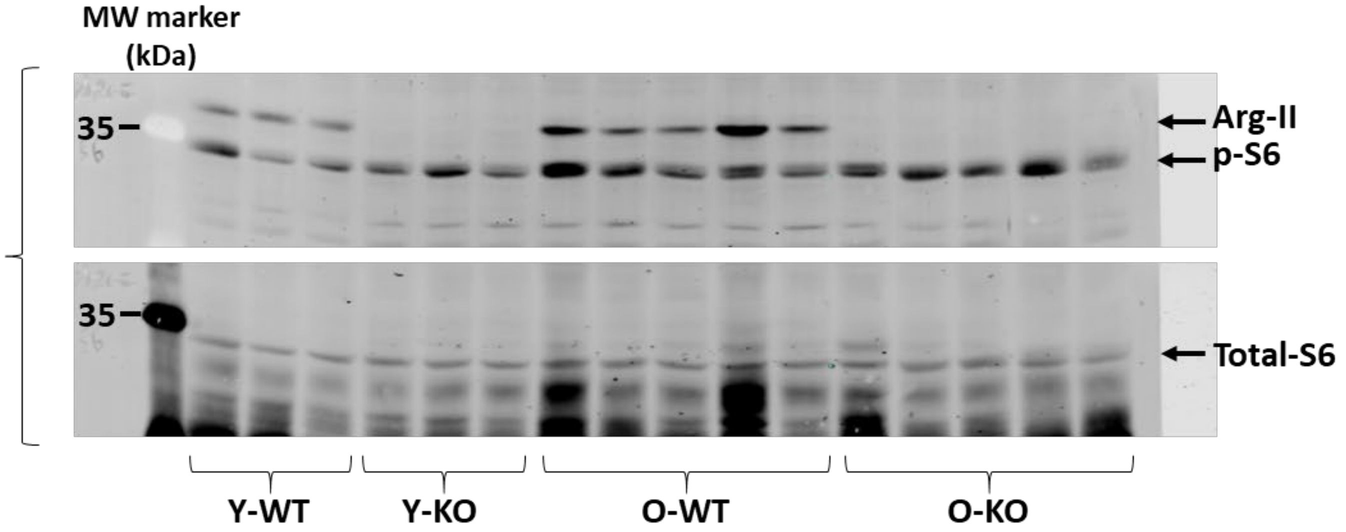

Suppl Fig.5d Female

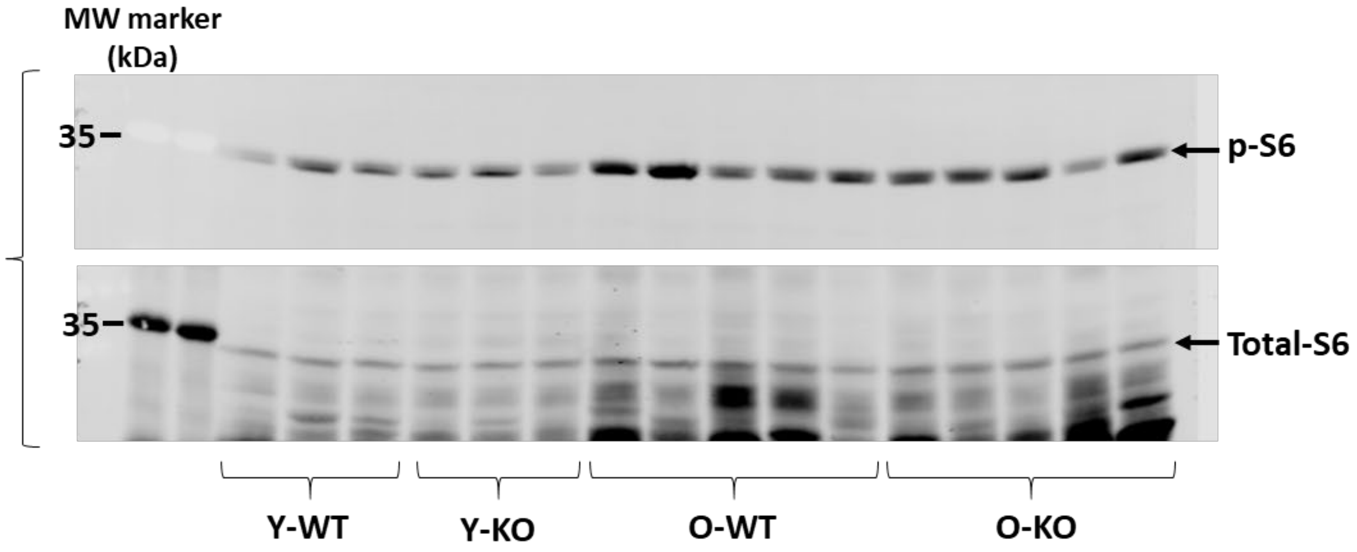

Procedure for processing Masson Trichrome images with ImageJ

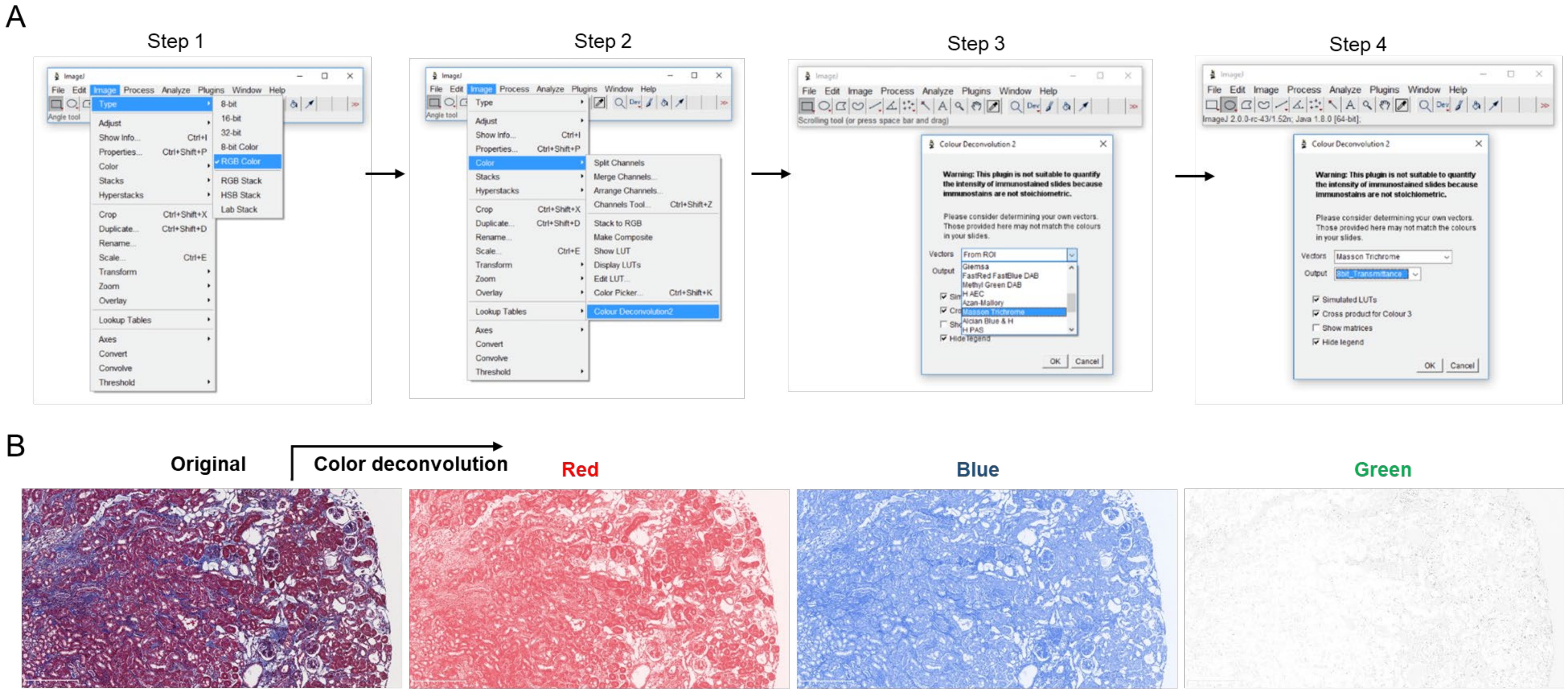

Image processed by color deconvolution. (A) The image was converted to an RGB color space and processed using the "Color Deconvolution" (B) After Color deconvolution, the original image was split into red, blue and green components.

Procedure for processing Masson Trichrome images with ImageJ

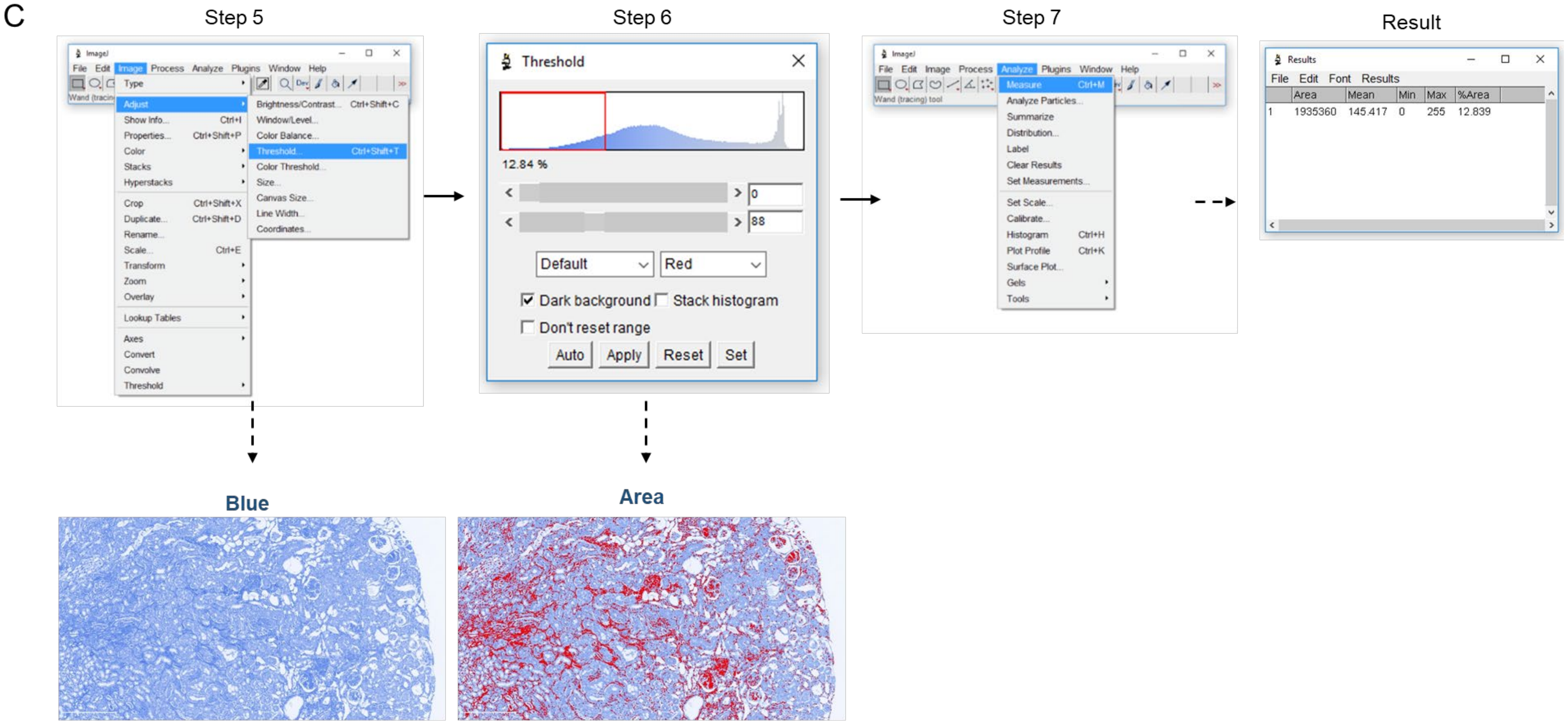

Quantification of the blue component using ImageJ software. (C) The threshold (step 5) was set to a constant value (step 6) for blue component. The result was shown as the percentage of positive area .
