# Supplementary material for: Role of tubular epithelial arginase-II in renal inflammaging
Source: NPJ Aging Mech Dis. 2021 Mar 2;7:5. doi: 10.1038/s41514-021-00057-8 (PMC7925687; doi:10.1038/s41514-021-00057-8)
Supplement: Supplementary file 2 — nr-reporting-summary [file 41514_2021_57_MOESM2_ESM.pdf]

nature research

Corresponding author(s): Zhihong YANG

Last updated by author(s): 24-11-2020

Reporting Summary

Nature Research wishes to improve the reproducibility of the work that we publish. This form provides structure for consistency and transparency in reporting. For further information on Nature Research policies, see our Editorial Policies and the Editorial Policy Checklist.

Statistics

For all statistical analyses, confirm that the following items are present in the figure legend, table legend, main text, or Methods section.

n/a	Confirmed	
☐	☒	The exact sample size (*n*) for each experimental group/condition, given as a discrete number and unit of measurement
☐	☒	A statement on whether measurements were taken from distinct samples or whether the same sample was measured repeatedly
☐	☒	The statistical test(s) used AND whether they are one- or two-sided<br>*Only common tests should be described solely by name; describe more complex techniques in the Methods section.*
☒	☐	A description of all covariates tested
☐	☒	A description of any assumptions or corrections, such as tests of normality and adjustment for multiple comparisons
☐	☒	A full description of the statistical parameters including central tendency (e.g. means) or other basic estimates (e.g. regression coefficient) AND variation (e.g. standard deviation) or associated estimates of uncertainty (e.g. confidence intervals)
☒	☐	For null hypothesis testing, the test statistic (e.g. *F*, *t*, *r*) with confidence intervals, effect sizes, degrees of freedom and *P* value noted<br>*Give P values as exact values whenever suitable.*
☒	☐	For Bayesian analysis, information on the choice of priors and Markov chain Monte Carlo settings
☒	☐	For hierarchical and complex designs, identification of the appropriate level for tests and full reporting of outcomes
☒	☐	Estimates of effect sizes (e.g. Cohen's *d*, Pearson's *r*), indicating how they were calculated

Our web collection on statistics for biologists contains articles on many of the points above.

Software and code

Policy information about availability of computer code

Data collection	Bio-Rad iQ5 software for Real time PCR, Image studio software for western blots, LAS X software for confocal imaging
Data analysis	NIH Image J software, Prism 6(GraphPad)

For manuscripts utilizing custom algorithms or software that are central to the research but not yet described in published literature, software must be made available to editors and reviewers. We strongly encourage code deposition in a community repository (e.g. GitHub). See the Nature Research guidelines for submitting code & software for further information.

Data

Policy information about availability of data

All manuscripts must include a data availability statement. This statement should provide the following information, where applicable:

- Accession codes, unique identifiers, or web links for publicly available datasets
- A list of figures that have associated raw data
- A description of any restrictions on data availability

The datasets that support the findings of this study are available from the corresponding author upon reasonable request.

Field-specific reporting

Life sciences study design

All studies must disclose on these points even when the disclosure is negative.

Sample size	No statistical method was used to determine the sample size. Based on our previous published studies, the sample size used in the present study was adequately powered to detect the observed differences between the experimental groups.
Data exclusions	No data were excluded .
Replication	Replication of the experimental findings generated similar results.
Randomization	Mice were not randomly assigned because we need to know the genotype and age of each mouse.
Blinding	Analysis of the mice was not blinded because we needed to know which group each mouse belonged to in order to do the analyses.

Reporting for specific materials, systems and methods

We require information from authors about some types of materials, experimental systems and methods used in many studies. Here, indicate whether each material, system or method listed is relevant to your study. If you are not sure if a list item applies to your research, read the appropriate section before selecting a response.

Materials & experimental systems

n/a	Involved in the study	
☐	☒	Antibodies
☐	☒	Eukaryotic cell lines
☒	☐	Palaeontology and archaeology
☐	☒	Animals and other organisms
☒	☐	Human research participants
☒	☐	Clinical data
☒	☐	Dual use research of concern

Methods

n/a	Involved in the study	
☒	☐	ChIP-seq
☒	☐	Flow cytometry
☒	☐	MRI-based neuroimaging

Antibodies

Antibodies used	The source of the antibodies used for immunoblotting and immunofluorescence staining were as followed: Arg-II (Cell Signaling, cat#55003), IL-1β (Abcam, cat#9722), MCP-1 (Abcam, cat#25124), VCAM-1 (Abcam, cat#134047), F4/80 (Cell Signaling, cat#30325S), ACE1 (Santa Cruz Biotechnology, cat#23908), TGF-β1 (Abcam, cat#215715), PCNA (Cell Signaling, cat#13110), p16 (Santa Cruz Biotechnology, cat#81156), Phospho-p70 S6 Kinase-Thr389 (Cell Signaling, cat#9234), ICAM-1 (Santa Cruz Biotechnology, cat#8439), S6 (Cell Signaling, cat#2317s), phospho S6-S240/244 (Cell Signaling, cat#5364), β-Actin(Sigma-Aldrich, cat#5441). The lot of numbers of each of the antibodies are unavailable.
Validation	The antibodies were purchased commercially based on the literature citations. All the antibodies used were validated either by the manufacturer's datasheet information or by information in the studies published in the literature. In addition, the antibodies used for western blotting were validated by the presence of a band at the expected molecular weight with reproducible results and by specific gene knockout/silencing. The antibodies used for immunofluorescence staining were validated by replacing primary antibody with IgG.

Eukaryotic cell lines

Policy information about cell lines

Cell line source(s)	HK2 cells were obtained from the American Type Culture Collection ATCC®CRL-2190™
Authentication	The cell line used was not authenticated.
Mycoplasma contamination	The cell line was negative for mycoplasma contamination.
Commonly misidentified lines (See ICLAC register)	None

Animals and other organisms

Policy information about studies involving animals; ARRIVE guidelines recommended for reporting animal research

Laboratory animals	C57BL6/J, females, males, Young (7-8 months) and old (24-28 months)

Wild animals	The study did not involve wild animals.
Field-collected samples	This study did not involve field-collected samples.
Ethics oversight	All animal protocols were approved by the Ethical Committee of Veterinary Office of Fribourg Switzerland.

Note that full information on the approval of the study protocol must also be provided in the manuscript.
